# Carabid beetles dataset from the Parco Regionale di Paneveggio e Pale di S. Martino (Dolomites: Italian Alps)

**DOI:** 10.3897/BDJ.12.e127417

**Published:** 2024-08-14

**Authors:** Roberto Pizzolotto

**Affiliations:** 1 Università della Calabria, Rende (CS), Italy Università della Calabria Rende (CS) Italy

**Keywords:** eastern Alps, extreme environments, insect communities, Paneveggio Pale di S. Martino Regional Park

## Abstract

**Background:**

Carabid beetles are one of the several taxa useful as model organisms to study and monitor current ecosystems features, as well as environmental changes caused by global changes. Open data about these organisms are scarcely available. To fill this gap, a data table (Darwin Core formatted) was uploaded in GBIF database (https://doi.org/10.15468/rzmp6n). The dataset is the result of a pitfall trapping survey of carabid beetles living along an altitudinal bioclimatic gradient, in the Dolomites mountains within the protected area of the Regional Park "Paneveggio e Pale di S. Martino" (Trento, Italy). Investigated environments ranged from spruce forests to the extreme environments of high altitude, so to collect a dataset as complete as possible on carabid beetles harboured in this area.

**New information:**

The dataset included here is part of an initiative aimed at promoting the adoption of a formal structure for datasets on carabid beetles acquired by field surveys and to give open access to these data. This dataset gives the opportunity to test the effects of global change affecting the same area, within long-term surveys on carabid beetles. Furthermore, the availability of open data is intended to promote an ethical approach to ecological research under a social and scientific point of view, the first because it will avoid wasting public funds on repeating the same researches and the second because it will avoid recapturing new organisms in the same (or similar) environments by other researchers.

## Introduction

Mountain environments represent a sort of natural laboratory where, over relatively short distances, the characteristics of the main continental ecoregions are reproduced thanks to altitude shifting (Soldati 2010). Animal species, adapted to high-altitude environments, are particularly sensitive to climate change and provide a model for studying species distribution in response to the main factors affecting mountain ecosystems (Körner et al. 2007, Schmitt et al. 2009). Ecological data collected within Alpine areas offer insights into the present scenario and serve as a powerful tool for comparing it with historical data (Pizzolotto et al. 2014) in the context of climate change.

In mountain ecosystems, the soil layer is of paramount importance as a facilitative factor for the colonisation of rocky substrates, then for supporting climax vegetation. This is one of the reasons that studies on alpine soil macrofauna receive greater attention, but require a high research effort because mountain ecosystems present different facies, even within the same ecological landscape (Guerra et al. 2020, Steinwandter and Seeber 2023).

It would be desirable, therefore, to use a common model organism amongst different alpine areas to allow for significant comparison of data both geographically and temporally. To do so, data following the FAIR principles (Findable, Accessible, Interoperable, Re-usable, see https://force11.org/info/the-fair-data-principles/) are of paramount importance to provide valuable data for basic and applied ecological studies. Online resources on carabid beetles are not FAIR for the most part or are not available as online data (i.e. grey literature), at least for Italy.

Carabid beetles represent a useful model organism capable of meeting the FAIR principles, they have intensively studied from the ecological and adaptive point of view and their taxonomy is very clear (Thiele 1977, Kotze et al. 2011, Turin et al. 2022). They are affected by the variation of biotic and abiotic factors at the regional, ecological landscape and continental scale (Vanbergen et al. 2005, Brandmayr et al. 2013); moreover, they are cost effective for sampling and identification effort, making them good biological indicators and model organisms (Cajaiba et al. 2018, McGregor and Wahl 2020).

## General description

### Purpose

The main purpose is to give open access to a dataset as complete as possible and according to FAIR principles, on distribution and abundance of carabid beetles harboured in the protected area of the "Parco Paneveggio e Pale di S. Martino", located within the UNESCO World Heritage area of the Dolomite Mountains (Italian Alps).

### Additional information

This is the second open dataset for the Italian carabid fauna published via the GBIF, following the first on carabid beetles living in beech Mediterranean forests (Pizzolotto 2022). The dataset was partly used by Pizzolotto et al. (2014), Pizzolotto et al. (2016), where a comparison with the previous work of Brandmayr and Zetto Brandmayr (1988) was given and now it is published as an open access scientific resource within the GBIF database (https://doi.org/10.15468/rzmp6n), with the properties of being findable, accessible, interoperable and re-usable.

The study area was within the protected area of the Regional Park "Paneveggio e Pale di S. Martino" (Trento, Italy https://www.parcopan.org/) and included an altitudinal bioclimatic gradient of environments from spruce forests to high-altitude ecosystems, where extreme ecological conditions affect living organisms. The difference in altitude was from 1650 m a.s.l. to 2680 m a.s.l. For a detailed description of the sample sites, see Supplementary Materials.

The carabid beetles harboured by these ecosystems are of particular importance from a biogeographical point of view and because of their adaptation to living in harsh conditions. This is why some of the sampled species contribute to the peculiarity of the fauna living in the studied region, where only species adapted to and selected by high altitude extreme environments can survive. The most peculiar species are *Leistuspunctatissimus* Breit, 1914, *Nebriagermarii* Heer, 1837 and *Trechusdolomitanus* Jeannel, 1931, because they are stenoendemic species living mainly in environments above 2500 metres in altitude, scarcely covered by patches of vegetation. *Pterostichusschaschli* (Marseul, 1880) and *Amaraalpestris* A. & G.B.Villa, 1833, also contribute to characterise the endemic fauna, at lower altitudes. A detailed analysis of the dataset was given by Pizzolotto (2024b).

## Sampling methods

### Sampling description

Carabid beetles were collected between 2008 and 2014, sampling every year in different locations by pitfall traps. The altitudinal gradient from spruce forests to high altitudinal environments was followed (Boiti and Boiti Saffaro 1988, Pignatti-Wikus and Pignatti 1988), where the main environments characterising the ecological landscape were sampled (see Supplementary Materials and Pizzolotto (2024b)). At the end of every sampling campaign, a “year’s sample” was obtained, which is the sum of the collections made during the research campaign. The pitfall traps were plastic vessels with an upper diameter of 9.2 cm, a depth of 11 cm and a small opening at 4 cm below the border to avoid rainwater overflowing, filled with 200 ml of a conservative mixture made of wine vinegar saturated with table salt.

Given the unpredictable events that cause traps to break (e.g. cows, tourists, marmots), the number of individuals sampled in a year within each site (year sample) may be affected by an uneven sampling effort. For this reason, species abundance is evaluated as annual Activity Density (aAD), which is based on the total annual number of individuals caught and on the total annual sampling effort unit (EU), related to a period of ten days, as follows:

eu = (traps * days)/10

eu is the effort unit for each single sampling period, from which the total annual capture effort is given by the sum of:

EU = ∑eu

and, therefore,

aAD = total number of individuals / EU.

## Geographic coverage

### Description

**Figure 1.**
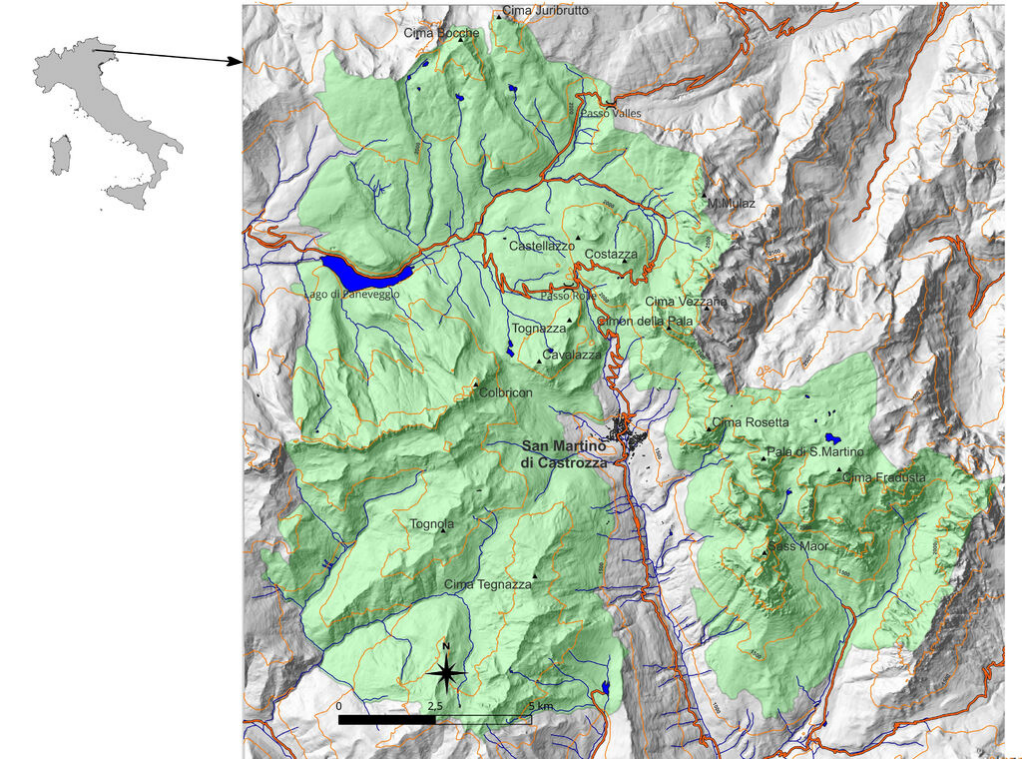
The study area was within the boundaries (green area) of the Parco di Paneveggio e Pale di S. Martino (Trento, Italy). For a detailed map with sample sites, see Supplementary Materials.

The study area was in Italy, in the administrative province of Trento, within the Dolomites, in the Regional Park "Paneveggio Pale di S. Martino". Detailed features of the sample sites are in Suppl. material 1 and in the "Dataset of carabid beetles in the Dolomite Mountains" (https://doi.org/10.15468/rzmp6n). See also Fig. 1.

### Coordinates

46.26304569 and 46.3530996 Latitude; 11.74784635 and 11.85632257 Longitude.

## Temporal coverage

**Data range:** 2008-6-30 – 2014-9-16.

### Notes

Various collection campaigns were carried out between 2008 and 2014; every year, different locations were sampled, as in Table 1.

## Usage licence

### Usage licence

Other

### IP rights notes

CC BY-NC 4.0

## Data resources

### Data package title

Dataset of carabid beetles in the Dolomite Mountains

### Resource link

https://doi.org/10.15468/rzmp6n

### Number of data sets

2

### Data set 1.

#### Data set name

event

#### Data format

Darwin Core table (tab delimited)

#### Description

In this dataset, the features of the 25 sample sites have been provided (Pizzolotto 2024a).

**Data set 1.** 

Column label	Column description
eventID	An identifier for the set of information associated with a dwc:Event (something that occurs at a place and time). Here an identifier specific to the dataset.
eventDate	The date-time or interval during which a dwc:Event occurred.
startDayOfYear	The earliest integer day of the year on which the dwc:Event occurred (e.g. 33 is the 2nd of February).
endDayOfYear	The latest integer day of the year on which the dwc:Event occurred.
habitat	A category or description of the habitat in which the dwc:Event occurred. Here the NAT2000 classification was used.
samplingProtocol	The names of, references to, or descriptions of the methods or protocols used during a dwc:Event.
sampleSizeValue	Number of active pitfall traps.
sampleSizeUnit	One pitfall trap.
samplingEffort	∑traps*(days/10).
countryCode	The standard code for the country in which the dcterms:Location occurs.
maximumElevationInMetres	The upper limit of the range of elevation (altitude, usually above sea level), in metres.
decimalLatitude	Latitude in decimal degrees.
decimalLongitude	Longitude in decimal degrees.
geodeticDatum	The ellipsoid, geodetic datum or spatial reference system (SRS), upon which the geographic coordinates given in dwc:decimalLatitude and dwc:decimalLongitude are based.
coordinateUncertaintyInMetres	The horizontal distance (in metres) from the given decimalLatitude and decimalLongitude describing the smallest circle containing the whole of the Location.
locality	Geographic information of the place where sample sites were located.

### Data set 2.

#### Data set name

occurrence

#### Data format

Darwin Core table (tab delimited)

#### Description

In this dataset, the abundances of the 45 carabid species sampled in 25 sites have been provided, for a total of 220 records (Pizzolotto 2024a).

**Data set 2.** 

Column label	Column description
basisOfRecord	The specific nature of the data record (here, human record).
occurrenceID	An identifier for the Occurrence, possibly a persistent global unique identifier. Here, it is given by the eventID plus the four first letters of genus and species.
individualCount	The number of individuals present at the time of the dwc:Occurrence.
organismQuantity	A number or enumeration value for the quantity of organisms. Here, an index of activity density (see organismQuantityType column).
organismQuantityType	The type of quantification system used for the quantity of organisms. Here, the activity density = individualCount/sampling effort.
lifeStage	The age class or life stage of the dwc:Organism(s) at the time the dwc:Occurrence was recorded.
occurrenceStatus	A statement about the presence or absence of a dwc:Taxon at a Location.
eventID	An identifier for the set of information associated with a dwc:Event (something that occurs at a place and time). Here an identifier specific to the dataset (the same as for the event table)
scientificName	The full scientific name.
kingdom	The full scientific name of the kingdom in which the species is classified.
phylum	The full scientific name of the phylum in which the species is classified.
class	The full scientific name of the class in which the species is classified.
order	The full scientific name of the order in which the species is classified.
family	The full scientific name of the family in which the species is classified.
genus	The full scientific name of the genus in which the species is classified.
specificEpithet	The name of the species epithet of the dwc:scientificName.
taxonRank	The taxonomic rank of the most specific name in the dwc:scientificName.

## Supplementary Material

DD7C6261-69B0-55AD-99E7-2D71F2ACE296 10.3897/BDJ.12.e127417.suppl1 Supplementary material 1 Sampled sites for the dataset on carabid beetles of the Regional Park (Paneveggio e Pale di San Martino, Italy) Data type topography, images File: oo_1046624.pdf https://binary.pensoft.net/file/1046624 Roberto Pizzolotto

## Figures and Tables

**Table:** 

Nanopublication	Creator	Date
https://w3id.org/kpxl/pensoft/bdj/np/RAChBolBOzhtbbk6E2EwOdKmnBbLselJ_udGkDTh-VlCI	Roberto Pizzolotto	08-07-2024 13:11:05
https://w3id.org/kpxl/pensoft/bdj/np/RA0_4sE7nouNkiR4XqH5Re5sw3ObGvVPSWMBhTI1mb0dk	Roberto Pizzolotto	08-07-2024 13:12:37
https://w3id.org/kpxl/pensoft/bdj/np/RATIF9BZ_eFROy7jdVzfMu0HNtZQtWYaOg4YskZGl0aKI	Roberto Pizzolotto	08-07-2024 13:13:57
https://w3id.org/kpxl/pensoft/bdj/np/RANv2fpeBRt05uDeTdD-Wbw1XvLxGSJJX_BfWFdpFOi0g	Roberto Pizzolotto	08-07-2024 13:15:28
https://w3id.org/kpxl/pensoft/bdj/np/RAo9K0SXCykYF3JiGvEGIP3l1oPJov9ynAig9sdVAbv7I	Roberto Pizzolotto	08-07-2024 13:17:21
https://w3id.org/kpxl/pensoft/bdj/np/RAqz49rNPaQmzMr9YjMkUoTaQ573REowL2DfTbVdDM0dY	Roberto Pizzolotto	08-07-2024 13:19:09
https://w3id.org/kpxl/pensoft/bdj/np/RAEwDEOluHzZrN5FUMO35TOtt5mUxUPfiGtwLaPsQ_I3Y	Roberto Pizzolotto	08-07-2024 13:20:31

**Table 1.** Sampled sites and date of sampling. More details in https://doi.org/10.15468/rzmp6n

eventID	eventDate
Alp1-2014	2014-07-22/2014-09-16
Alp2-2014	2014-07-22/2014-09-16
Alp3-2014	2014-07-22/2014-09-19
Alp4-2014	2014-08-24/2014-09-16
Cf1-2013-14	2014-07-21/2014-09-16
F1-2008	2008-06-30/2008-10-12
F2-2008	2008-06-30/2008-10-12
FH1-2012	2012-06-27/2012-09-08
FH2-2012	2012-06-27/2012-09-08
FH3-2012	2012-06-27/2012-09-08
L-2013	2013-06-30/2013-10-08
NA1-2009	2009-06-30/2009-09-26
NA2-2009	2009-07-01/2009-09-26
PS1-2008	2008-06-30/2008-10-12
PS2-2008	2008-06-30/2008-10-12
RM-2013	2013-06-30/2013-10-08
Rs-2013	2013-07-20/2013-08-29
Se1-2011	2011-06-29/2011-09-01
Se2-2011	2011-06-29/2011-09-01
Se3-2011	2011-06-29/2011-09-01
VN1-2013-14	2014-07-21/2014-09-16
VV1-2009	2009-07-01/2009-09-27
VV2-2009	2009-07-01/2009-09-27
VV3-2009	2009-07-01/2009-09-26
VV4-2009	2009-07-01/2009-09-27
