# Supplementary material for: Carabid beetles dataset from the Parco Regionale di Paneveggio e Pale di S. Martino (Dolomites: Italian Alps)
Source: Biodivers Data J. 2024 Aug 14;12:e127417. doi: 10.3897/BDJ.12.e127417 (PMC11342381; doi:10.3897/BDJ.12.e127417)

Carabid beetles of the Dolomites, a dataset from the Parco Regionale di Paneveggio e Pale di S.Martino (Italy)

Sampled sites

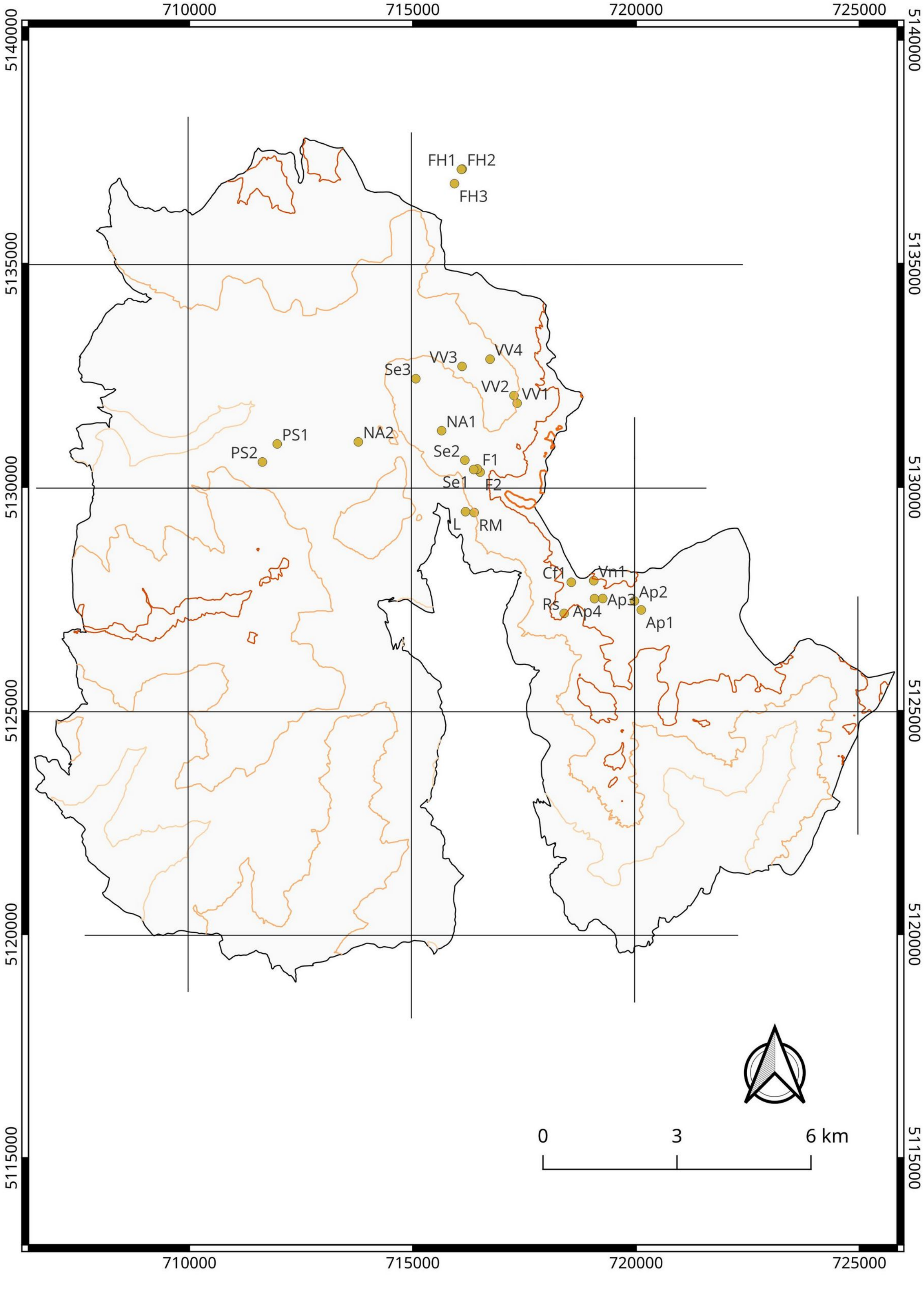

Black line: border of the Paneveggio Park; orange lines: altitudinal profile; frame (and grid): metric cohordinates following WGS84 - UTM32N system.

**PS1**	Subalpine spruce forest with dense blueberry undergrowth and soil rich in sphagnum moss. NAT2000: 9410				
altitude m a.s.l.	aspect	slope°	vegetation %	traps	year
1650	NW	10°	90%	5	2008

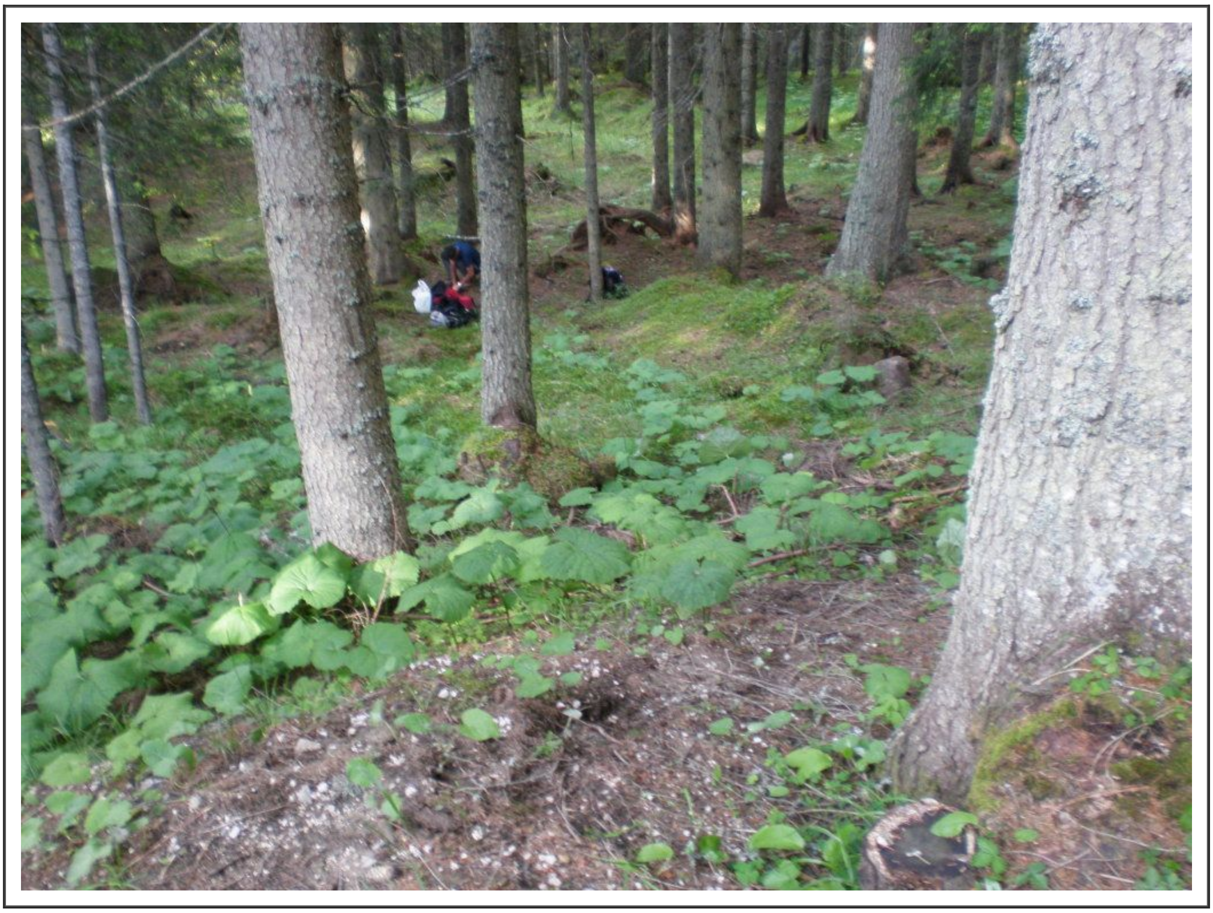

**PS2**	Subalpine spruce forest with blueberry undergrowth and soil with sphagnum moss. NAT2000: 9410				
altitude m a.s.l.	aspect	slope°	vegetation %	traps	year
1780	NNE	25°	65%	5	2008

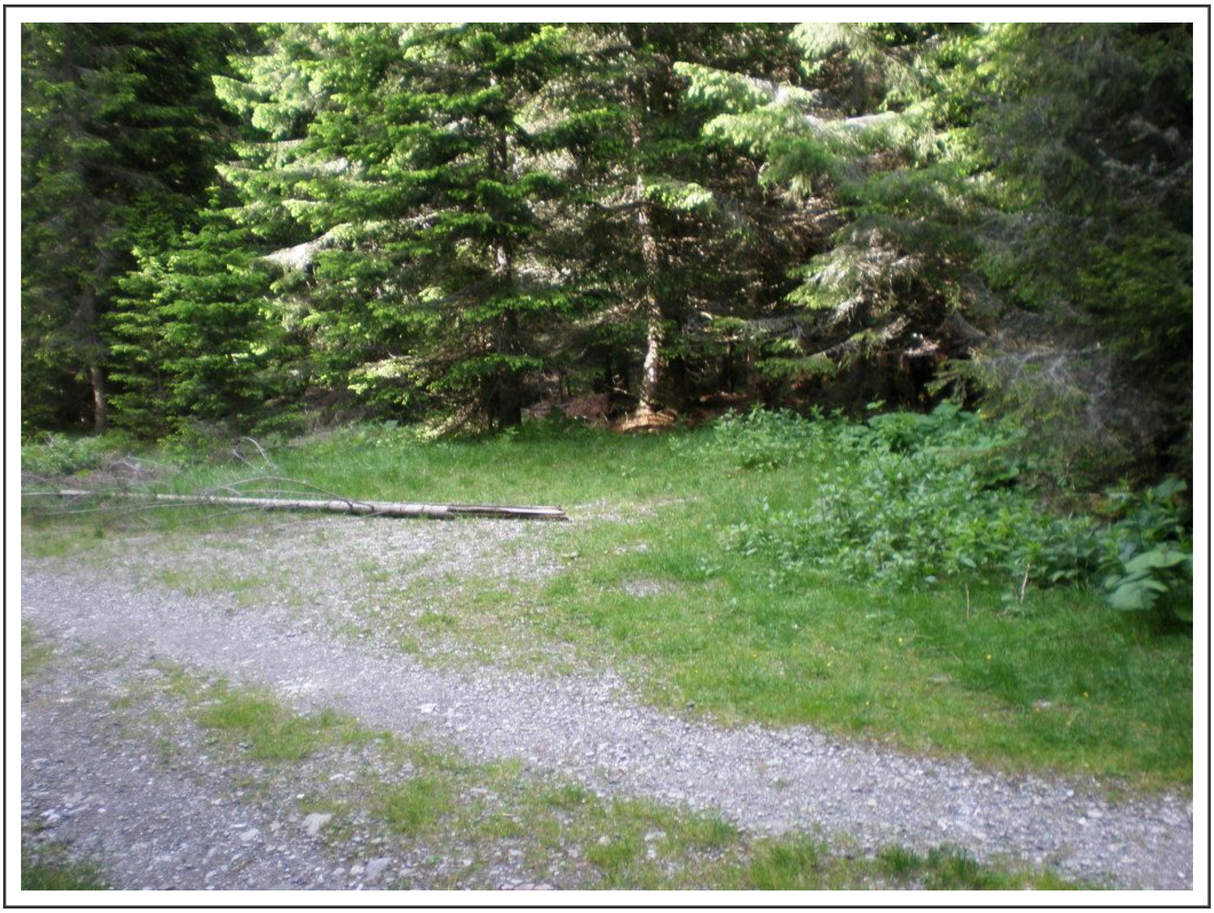

**VV3**	Mixed forest dominated by spruce. NAT2000: 9410				
altitude m a.s.l.	aspect	slope°	vegetation %	traps	year
1800	NW	30°	arborea 70%,	5	2009

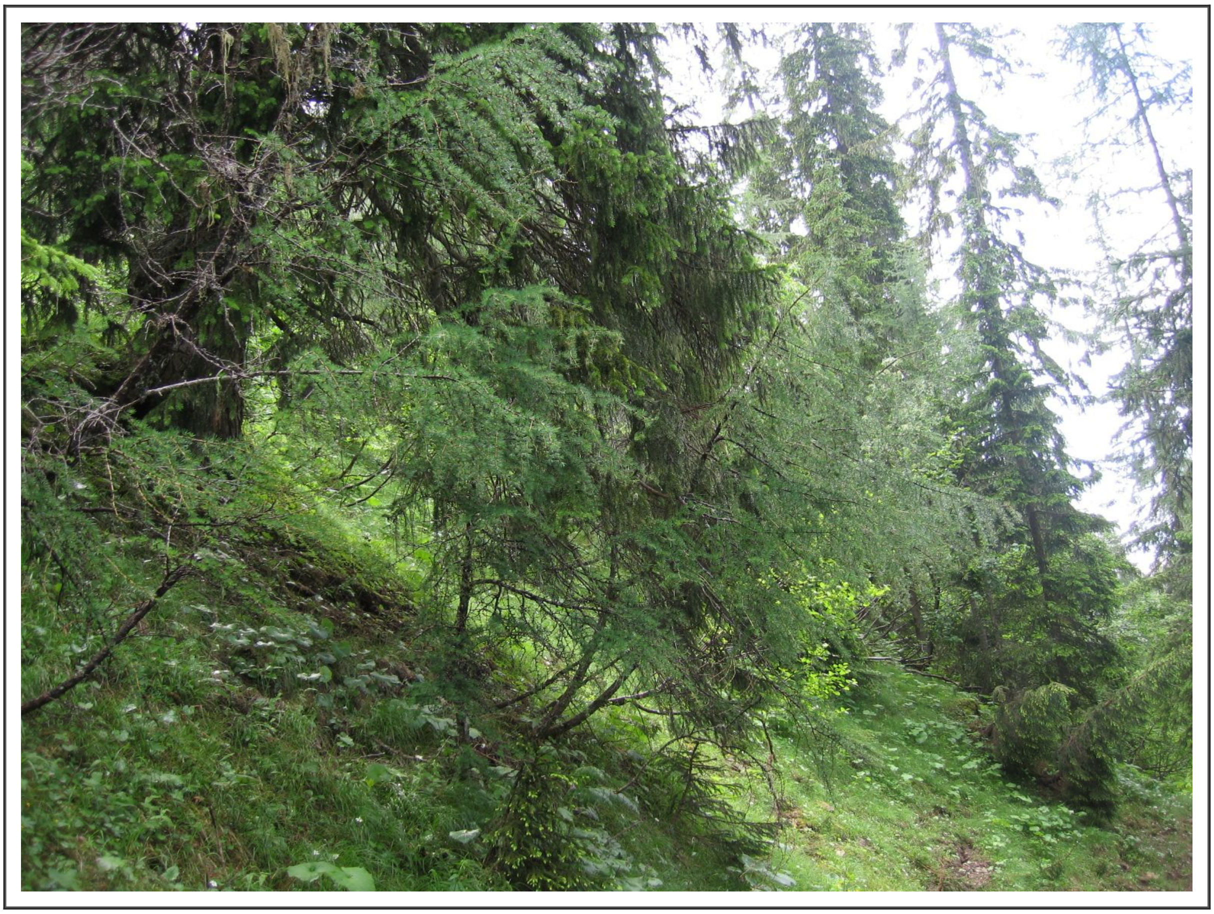

**VV4**	Man-planted spruce forest.				
altitude m a.s.l.	aspect	slope°	vegetation %	traps	year
1850	SE	40°	95%	5	2009

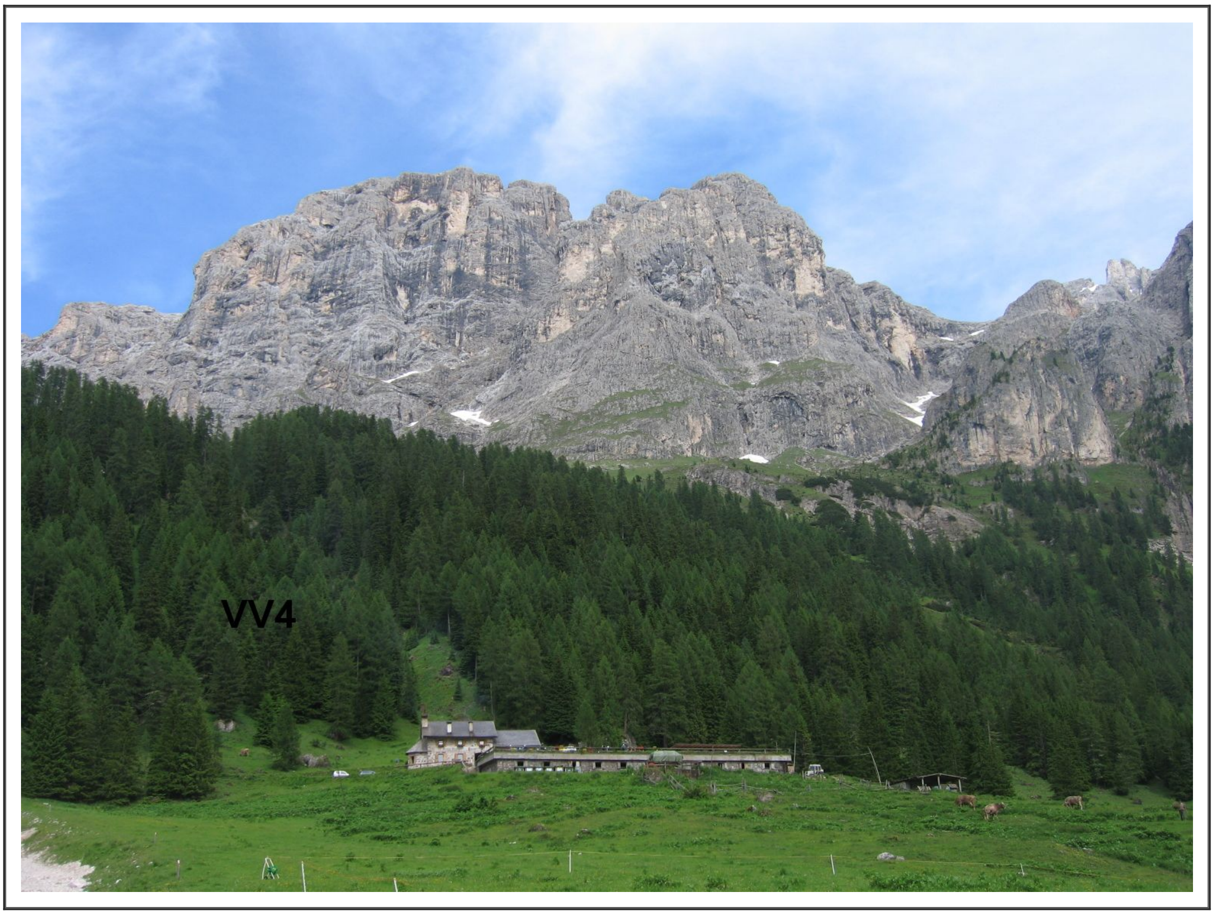

**VV2**	Vegetation with dwarf bushes and sparse willows, mainly Rhododendron, Dryas and blueberry. NAT2000: 4060, 4070				
altitude m a.s.l.	aspect	slope°	vegetation %	traps	year
1950	W	10°	50%	5	2009

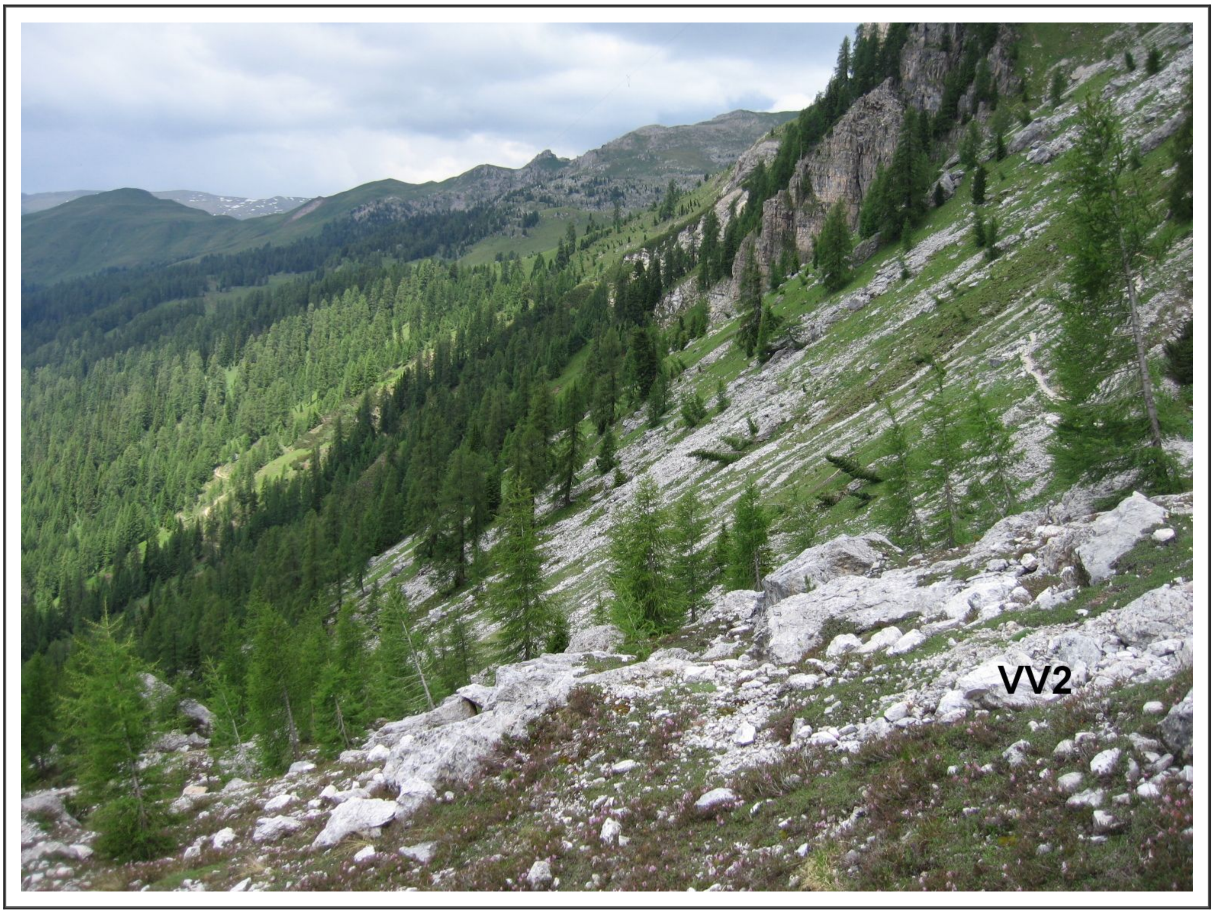

SE1	Alpine meadow with Sesleria varia and Carex sempervirens. NAT2000: 6170				
altitude m a.s.l.	aspect	slope°	vegetation %	traps	year
2000	N	5°	90%	6	2011

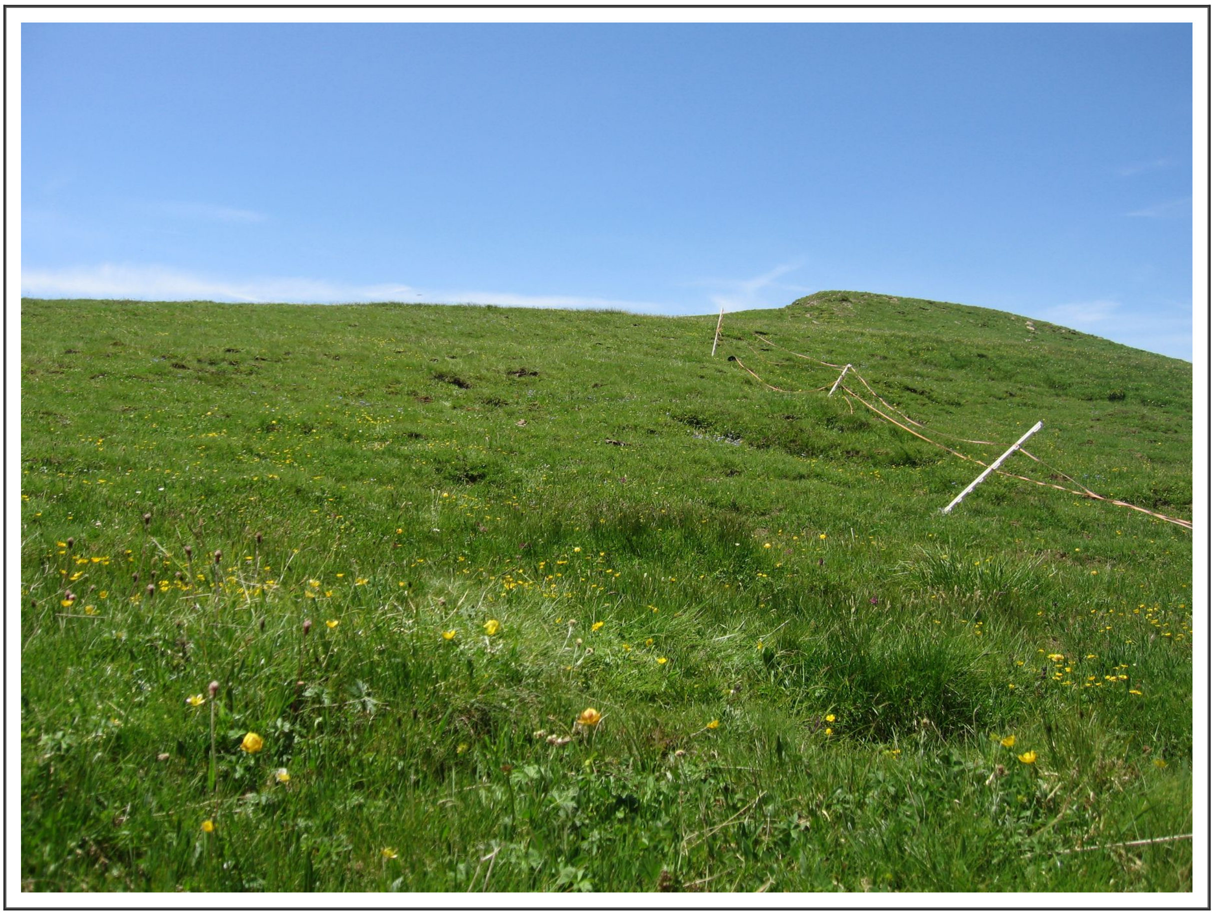

<table>
<tr><td>SE2</td><td colspan="5">Alpine meadow with Sesleria varia and Carex sempervirens. NAT2000: 6170</td></tr>
<tr><td>altitude m a.s.l.</td><td>aspect</td><td>slope°</td><td>vegetation %</td><td>traps</td><td>year</td></tr>
<tr><td>2000</td><td>N</td><td>20°</td><td>90%</td><td>6</td><td>2011</td></tr>
</table>

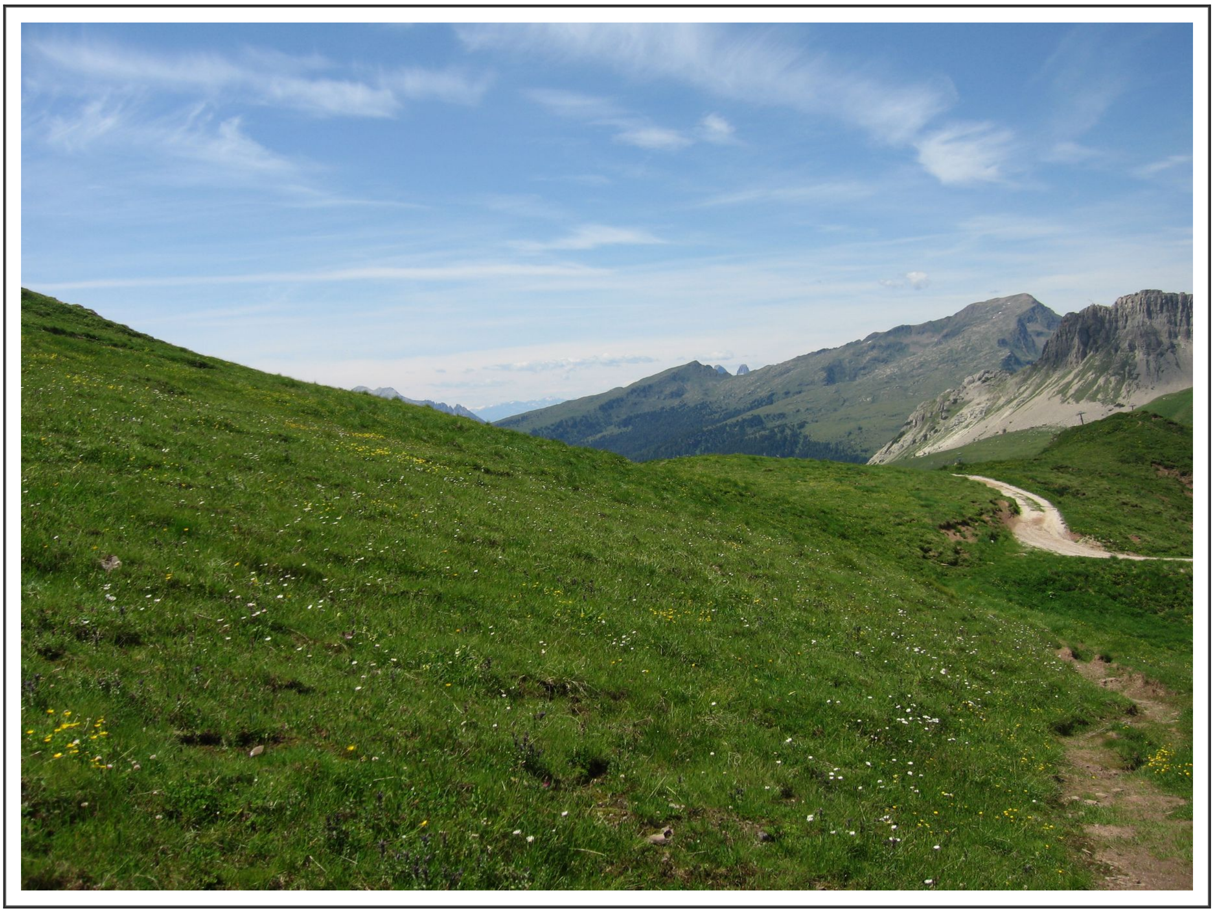

**SE3**	Alpine meadow with Sesleria varia and Carex sempervirens. NAT2000: 6170				
altitude m a.s.l.	aspect	slope°	vegetation %	traps	year
2000	N	15°	100%	6	2011

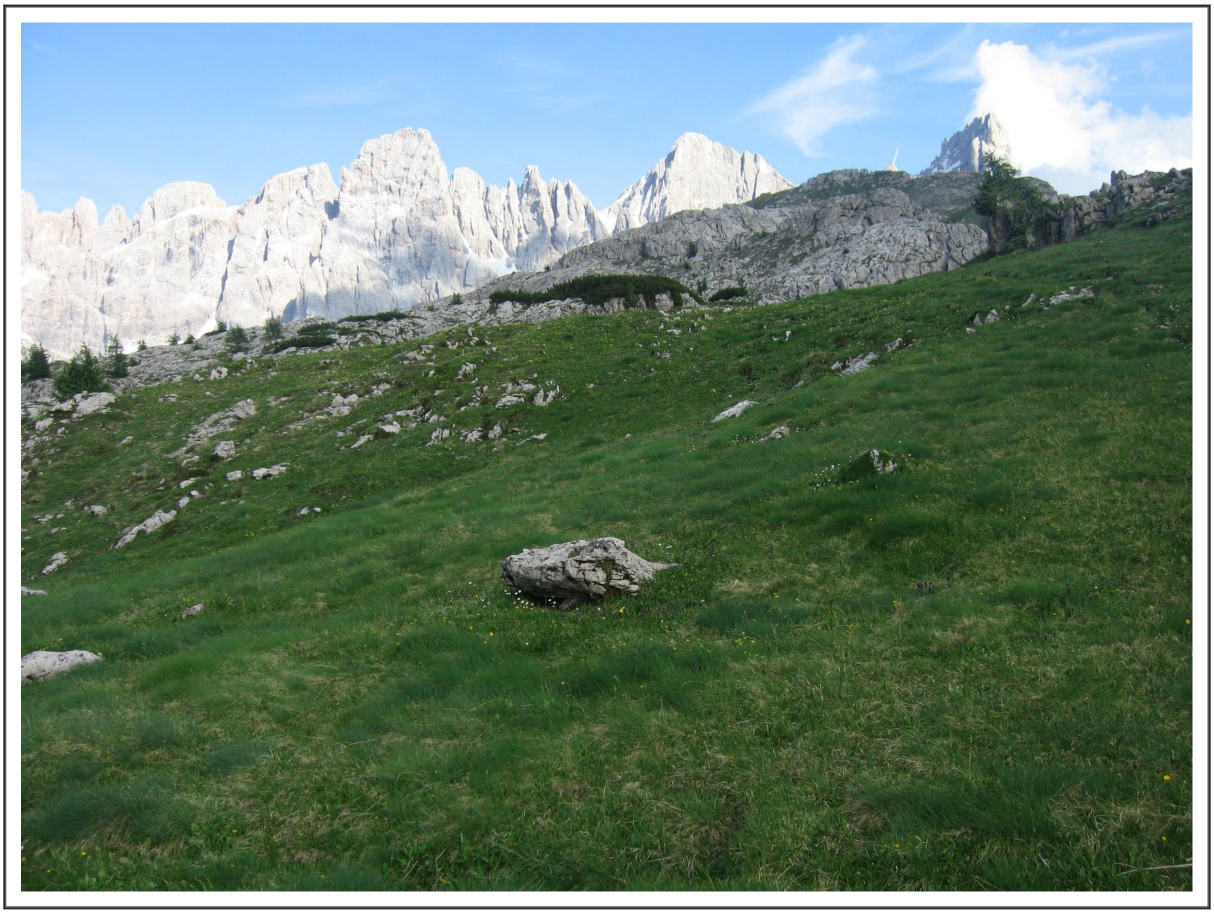

<table>
<tr><td>NA1</td><td colspan="5">Pasture of Nardus derived from trampling of Rhododendron moors. NAT2000: 6150</td></tr>
<tr><td>altitude m a.s.l.</td><td>aspect</td><td>slope°</td><td>vegetation %</td><td>traps</td><td>year</td></tr>
<tr><td>2170</td><td>SSW</td><td>20°</td><td>100%</td><td>6</td><td>2009</td></tr>
</table>

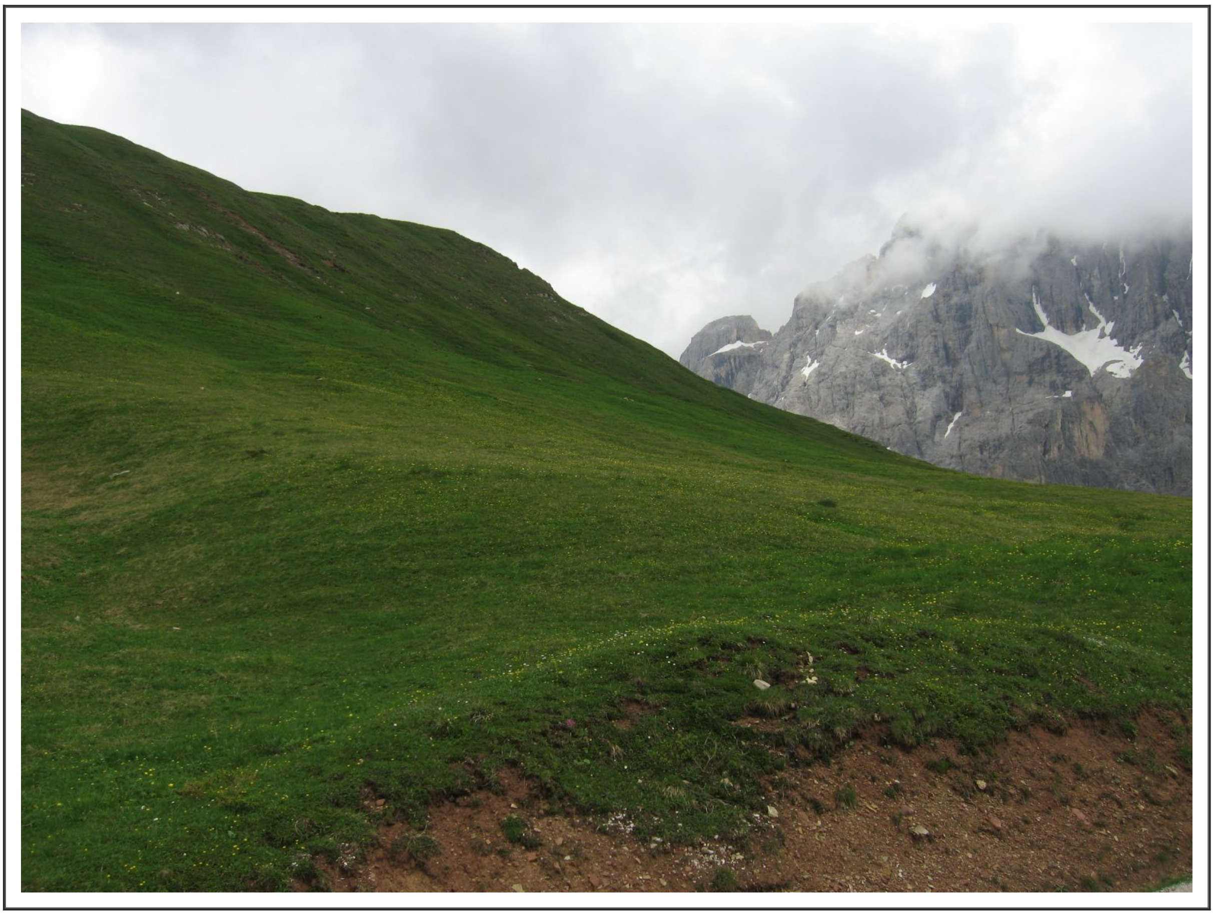

**NA2**	Pasture of Nardus resulting from the deforestation of a larch-cember wood. NAT2000: 6150				
altitude m a.s.l.	aspect	slope°	vegetation %	traps	year
1910	S	15°	100%	5	2009

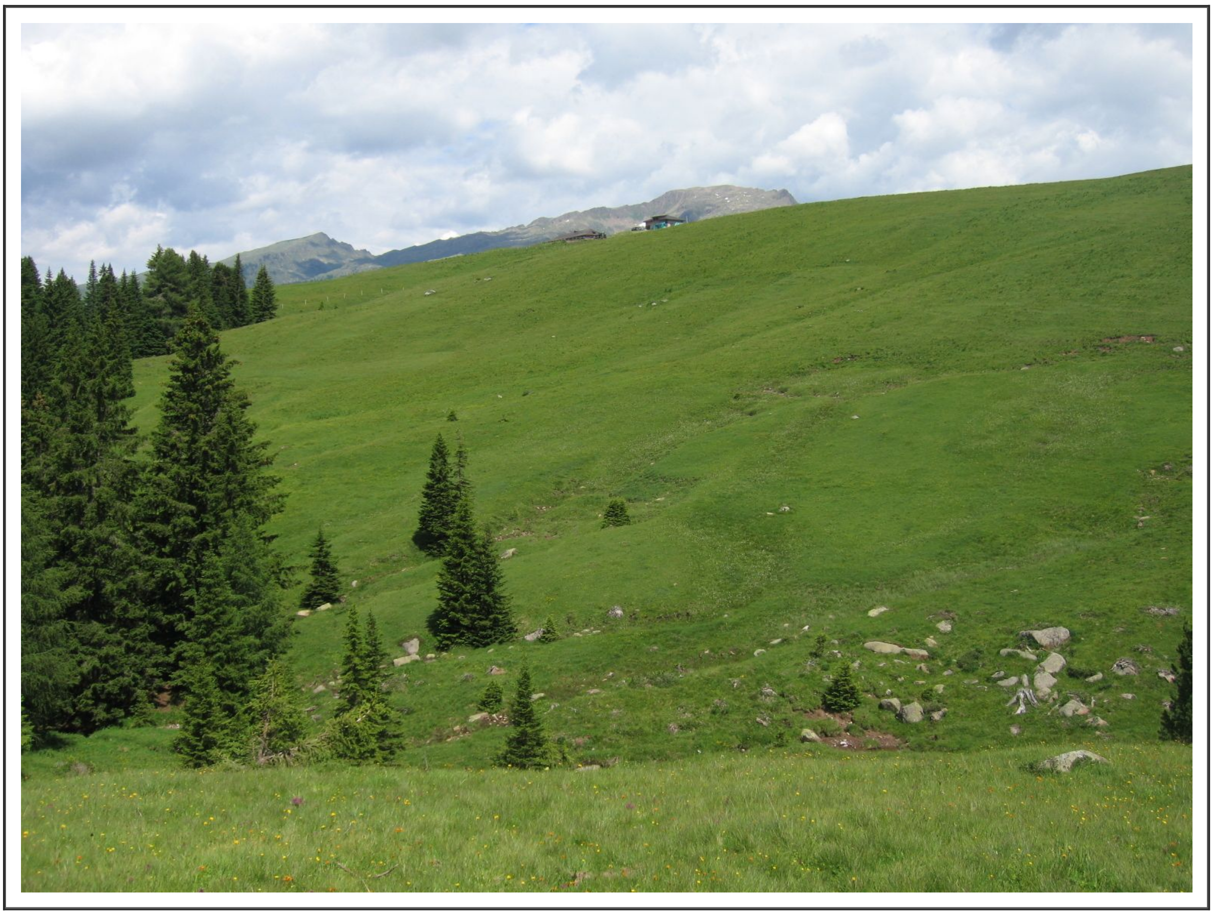

**F1**	Fragments of alpine meadow and scree vegetation. NAT2000: 8120				
altitude m a.s.l.	aspect	slope°	vegetation %	traps	year
2200	NNW	35°	60%.	5	2008

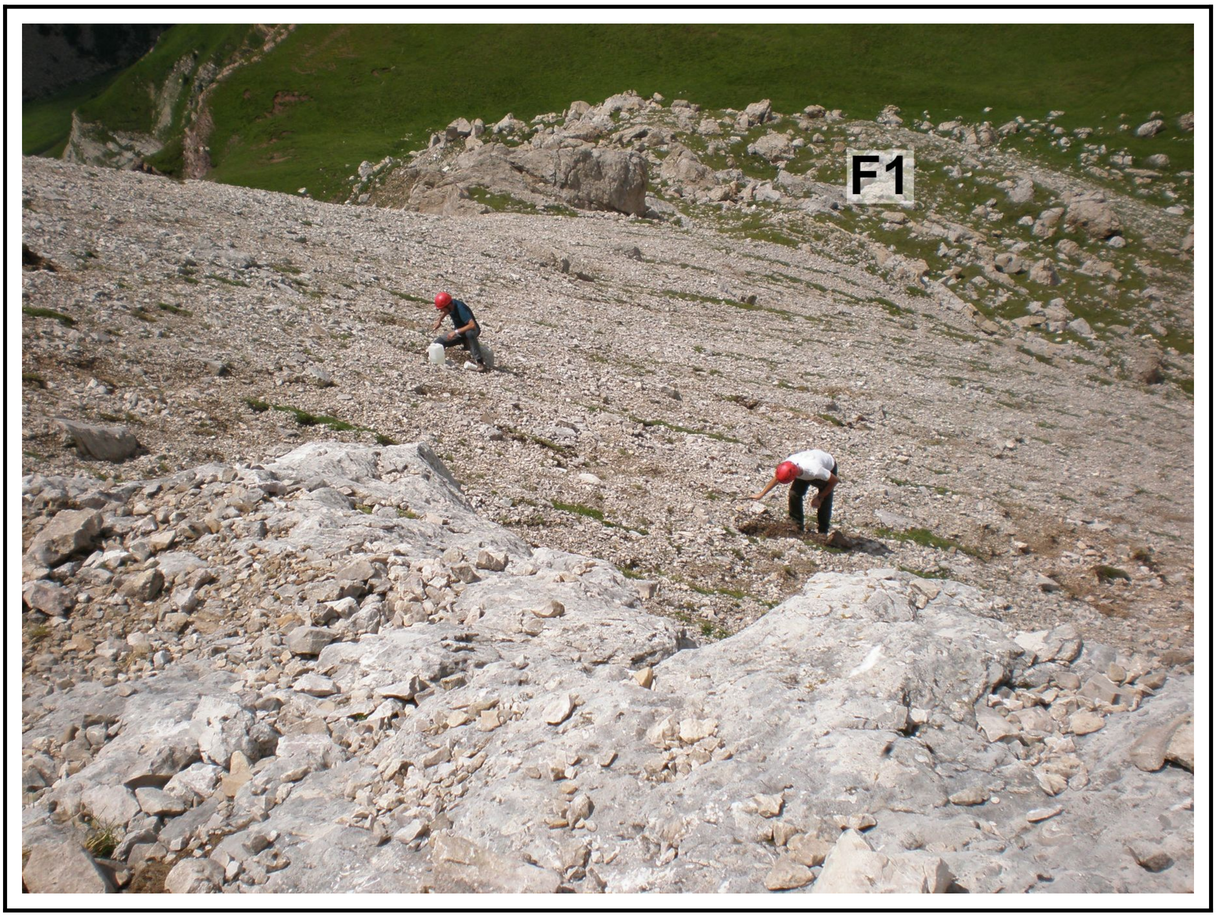

**F2**	Pioneer scree vegetation. NAT2000: 8120				
altitude m a.s.l.	aspect	slope°	vegetation %	traps	year
2250	NW	35°	20%	5	2008

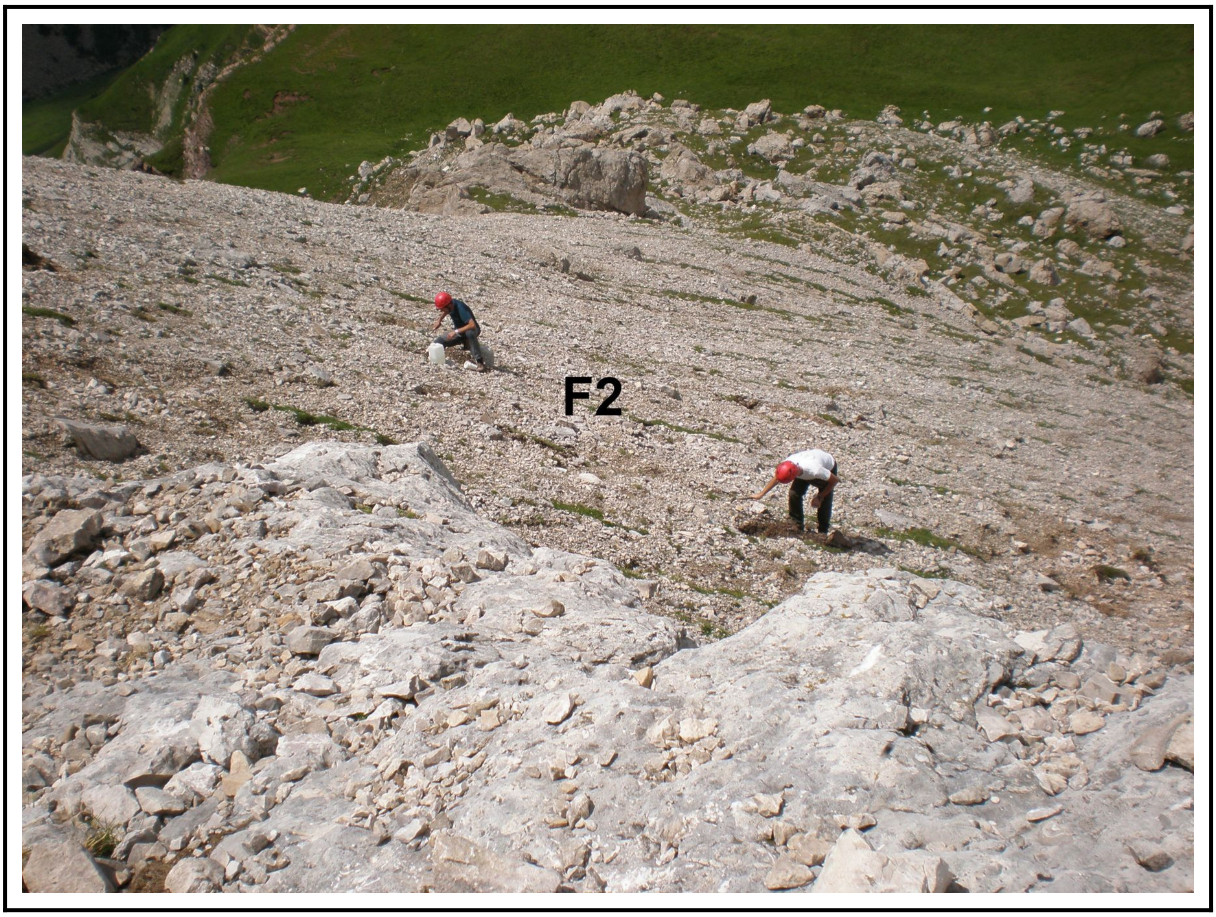

**VV1**	Pioneer scree vegetation. NAT2000: 8120				
altitude m a.s.l.	aspect	slope°	vegetation %	traps	year
2000	W	35°	10%.	5	2009

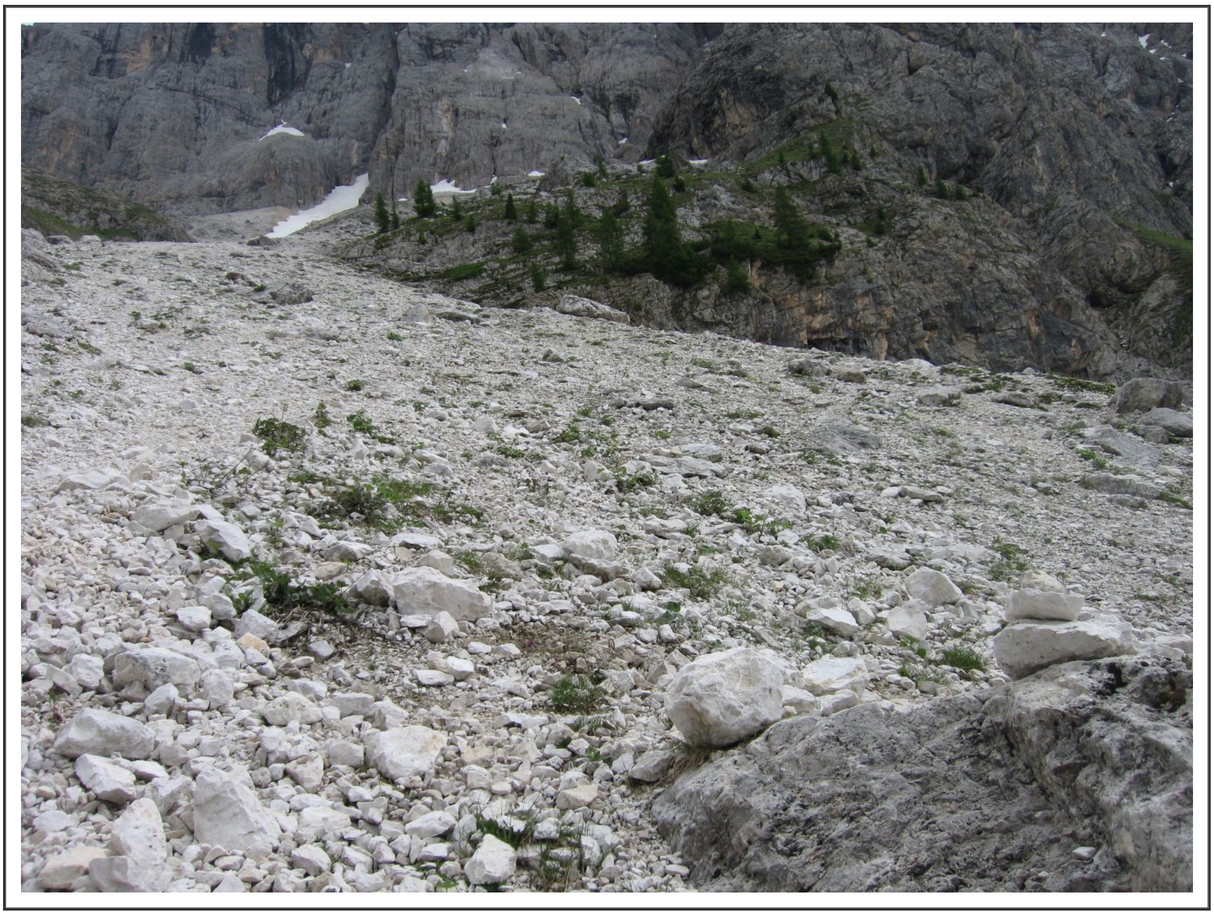

**L**	Larch forest with abundant Erica undergrowth. NAT2000: 9420				
altitude m a.s.l.	aspect	slope°	vegetation %	traps	year
1950	W	25°	Copertura Larice 45%	6	2013

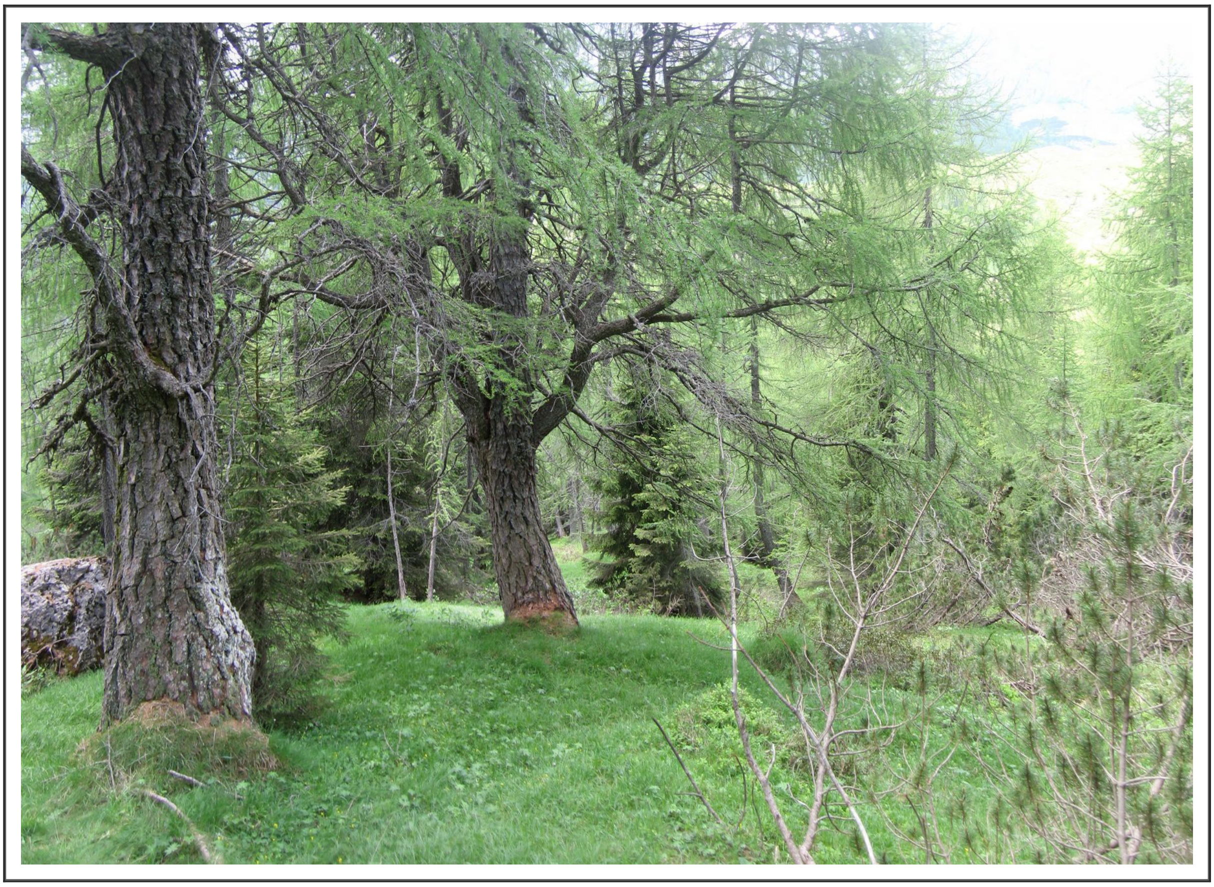

**RM**	Mountain pine forest. NAT2000: 4070				
altitude m a.s.l.	aspect	slope°	vegetation %	traps	year
1970	W	25	70	6	2013

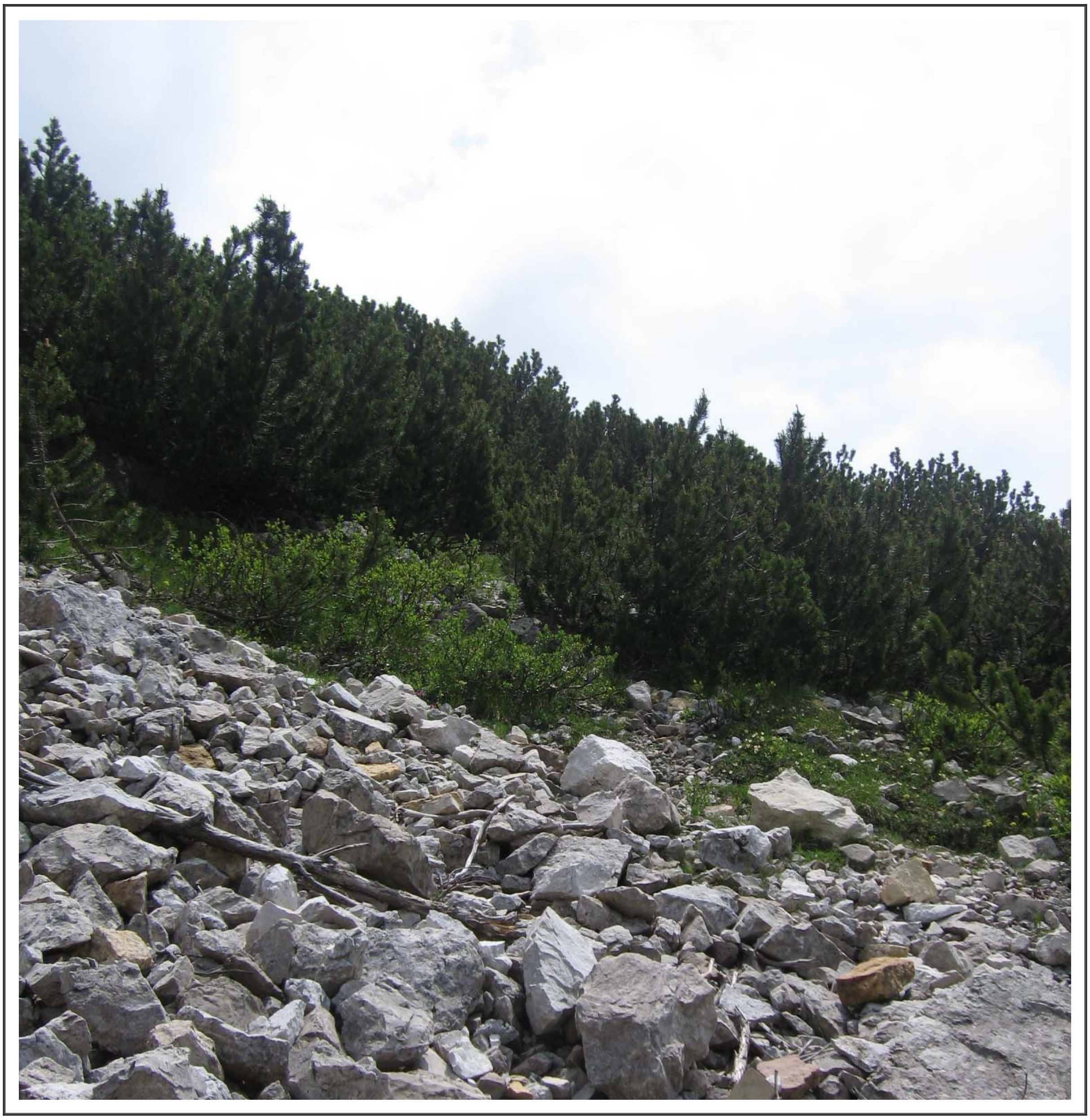

FH1	Acidophilic pasture of Festuca. NAT2000: 6150				
altitude m a.s.l.	aspect	slope°	vegetation %	traps	year
2230	WSW	8-12°	95%	6	2012

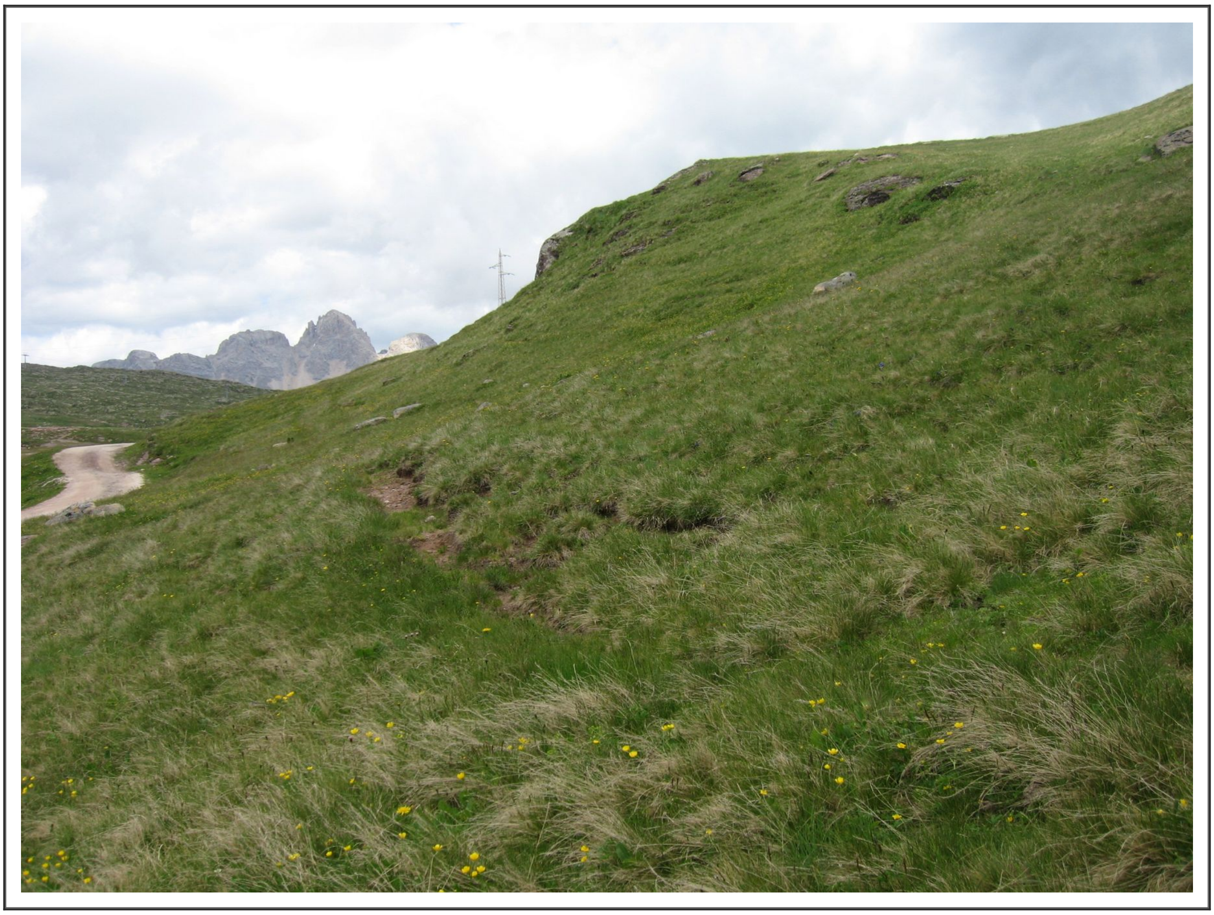

**FH2**	Acidophilic pasture of Festuca. NAT2000: 6150				
altitude m a.s.l.	aspect	slope°	vegetation %	traps	year
2245	WSW	25°	70%	6	2012

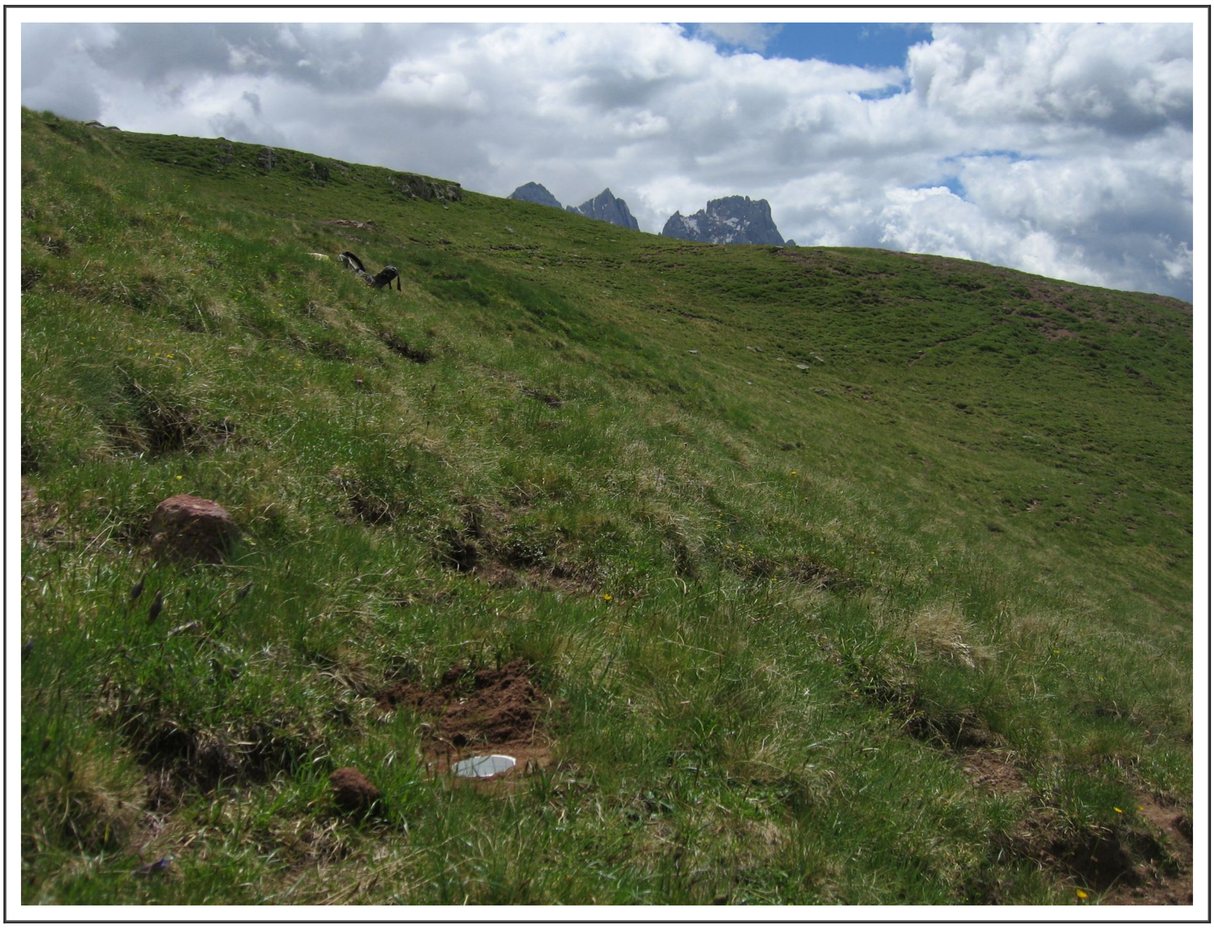

**FH3**	Acidophilic pasture of Festuca. NAT2000: 6150				
altitude m a.s.l.	aspect	slope°	vegetation %	traps	year
2175	WSW	5-7°	100%	6	2012

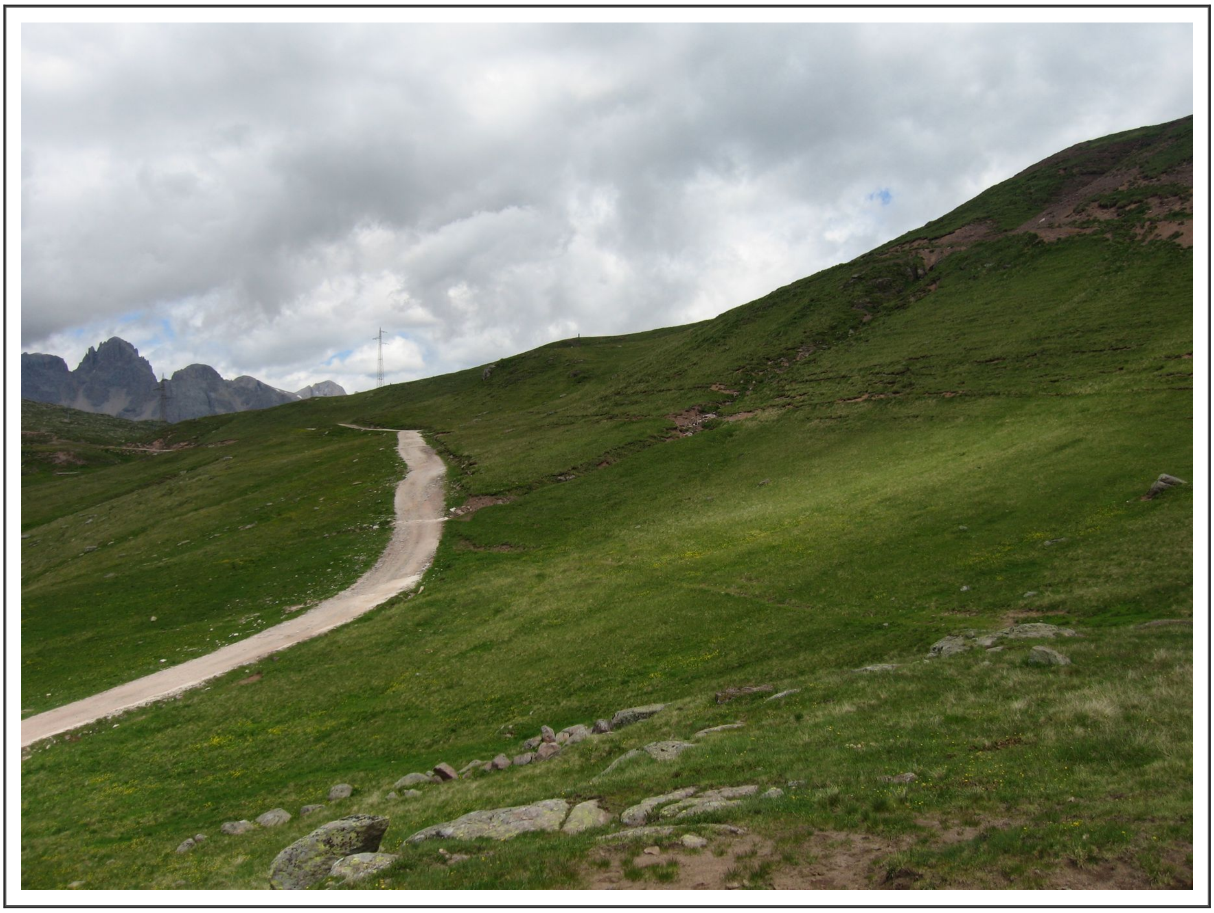

**VN1**	Snow bed. NAT2000: 6170				
altitude m a.s.l.	aspect	slope°	vegetation %	traps	year
2512	S	10°	80%	5	2013-14

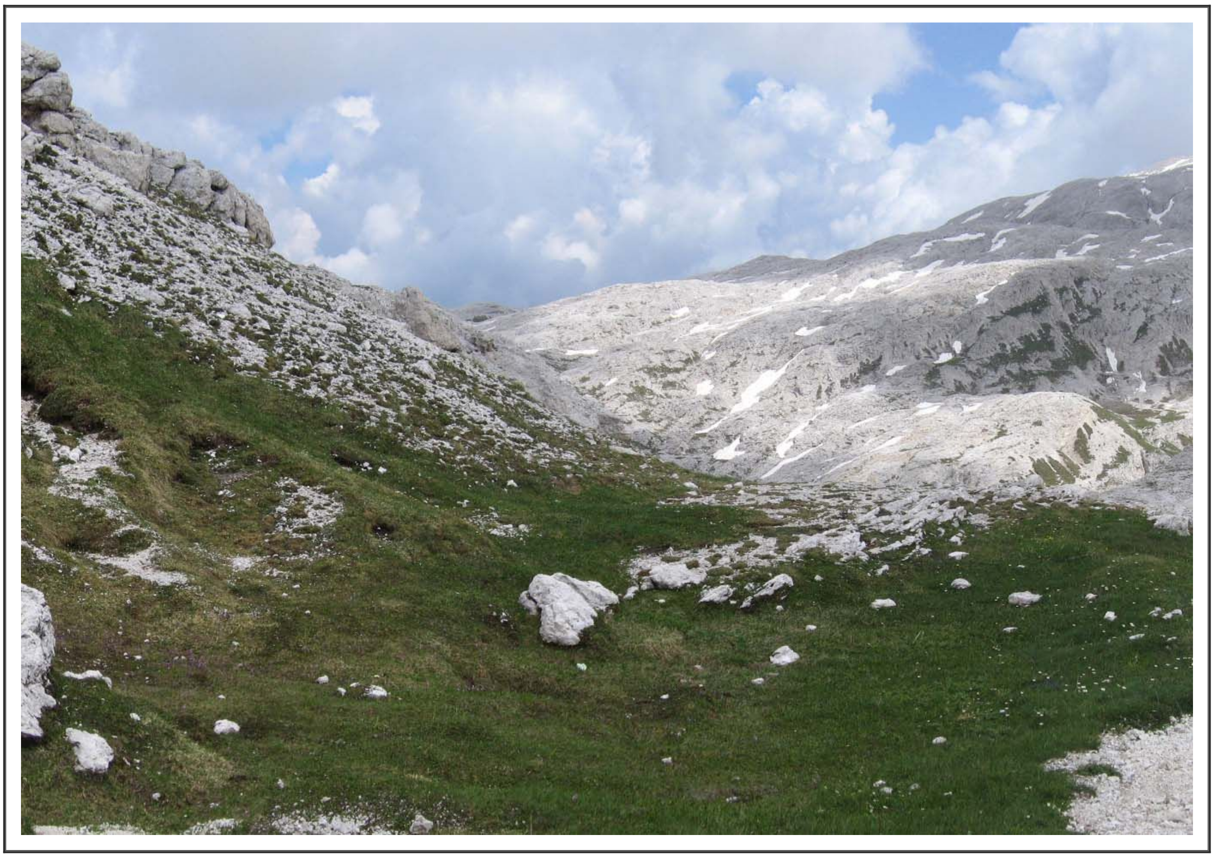

**ALP1**	Small patches of vegetation on limestone bedrock. NAT2000: 8240, 8120				
altitude m a.s.l.	aspect	slope°	vegetation %	traps	year
2650	N	10°	5%	6	2014

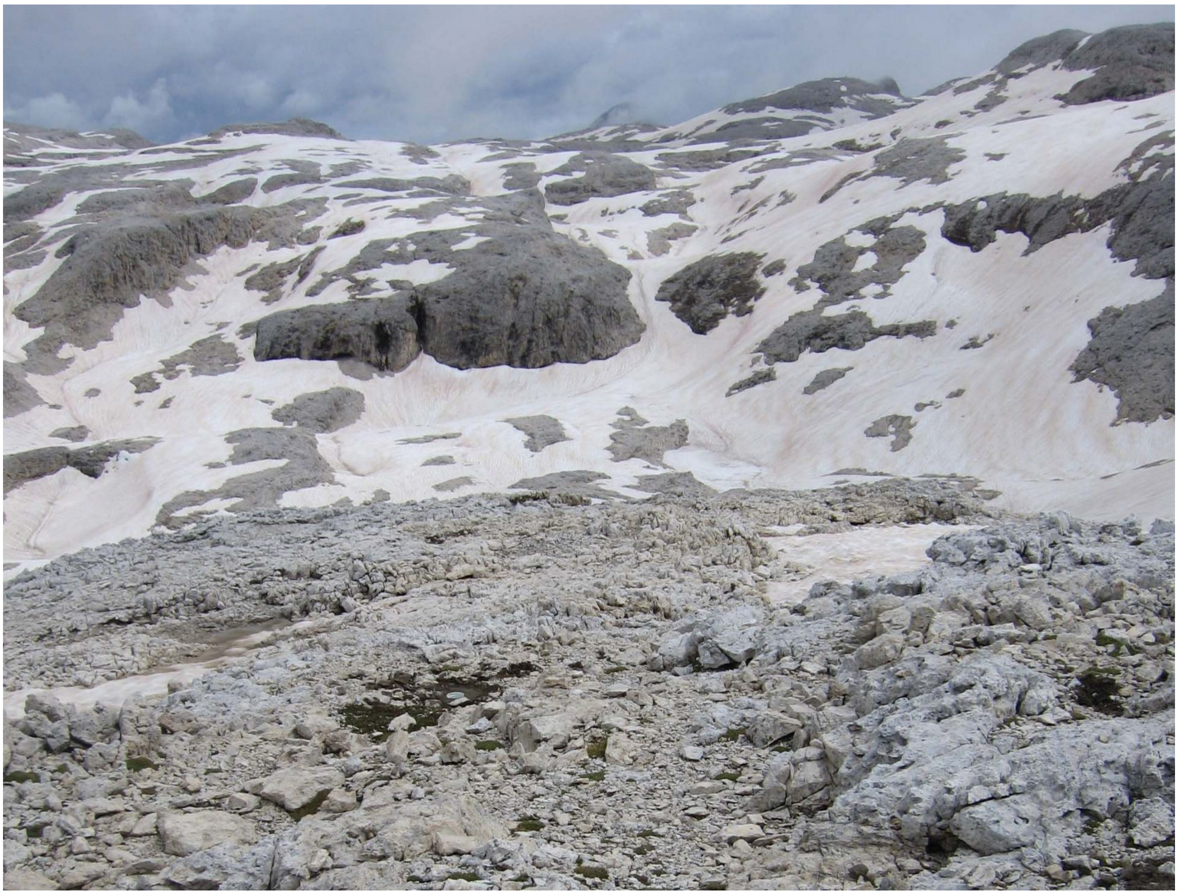

**ALP2**	Small patches of vegetation on limestone bedrock. NAT2000: 8240, 8120				
altitude m a.s.l.	aspect	slope°	vegetation %	traps	year
2625	S	25°	5%	6	2014

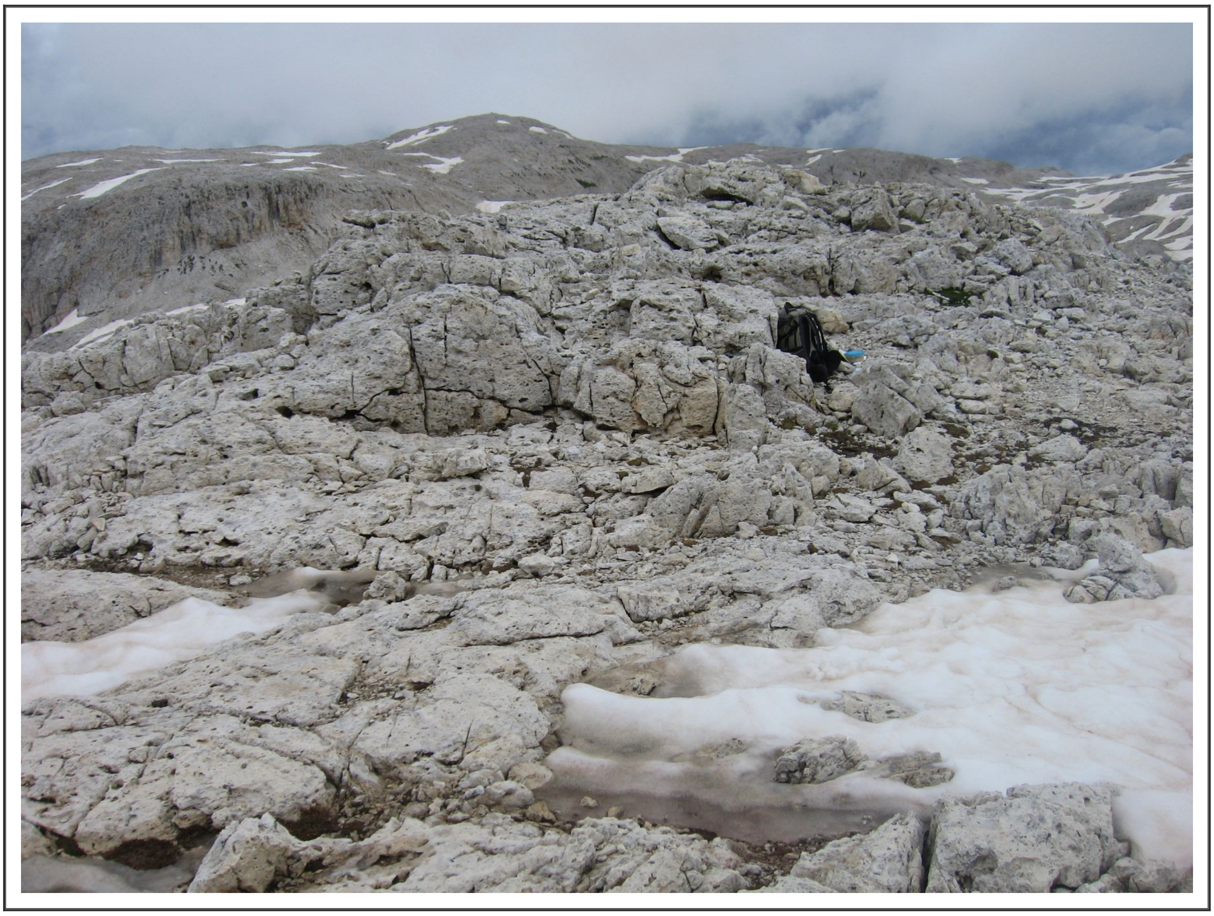

**ALP3**	Small patches of vegetation on limestone bedrock. NAT2000: 8240, 8120				
altitude m a.s.l.	aspect	slope°	vegetation %	traps	year
2550	E	25°	10%	6	2014

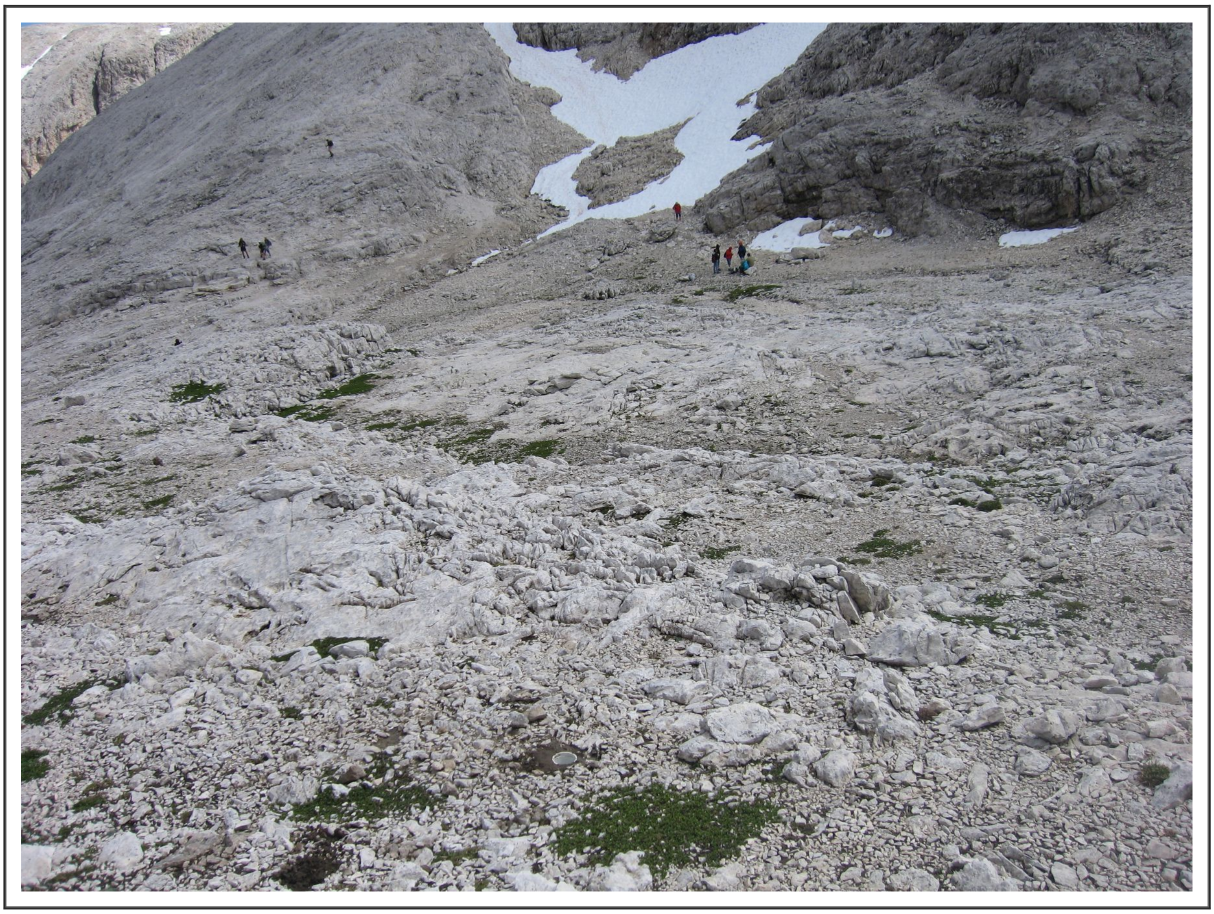

**ALP4**	Small patches of vegetation on limestone bedrock. NAT2000: 8240, 8120				
altitude m a.s.l.	aspect	slope°	vegetation %	traps	year
2550	E	10°	40%	5	2014

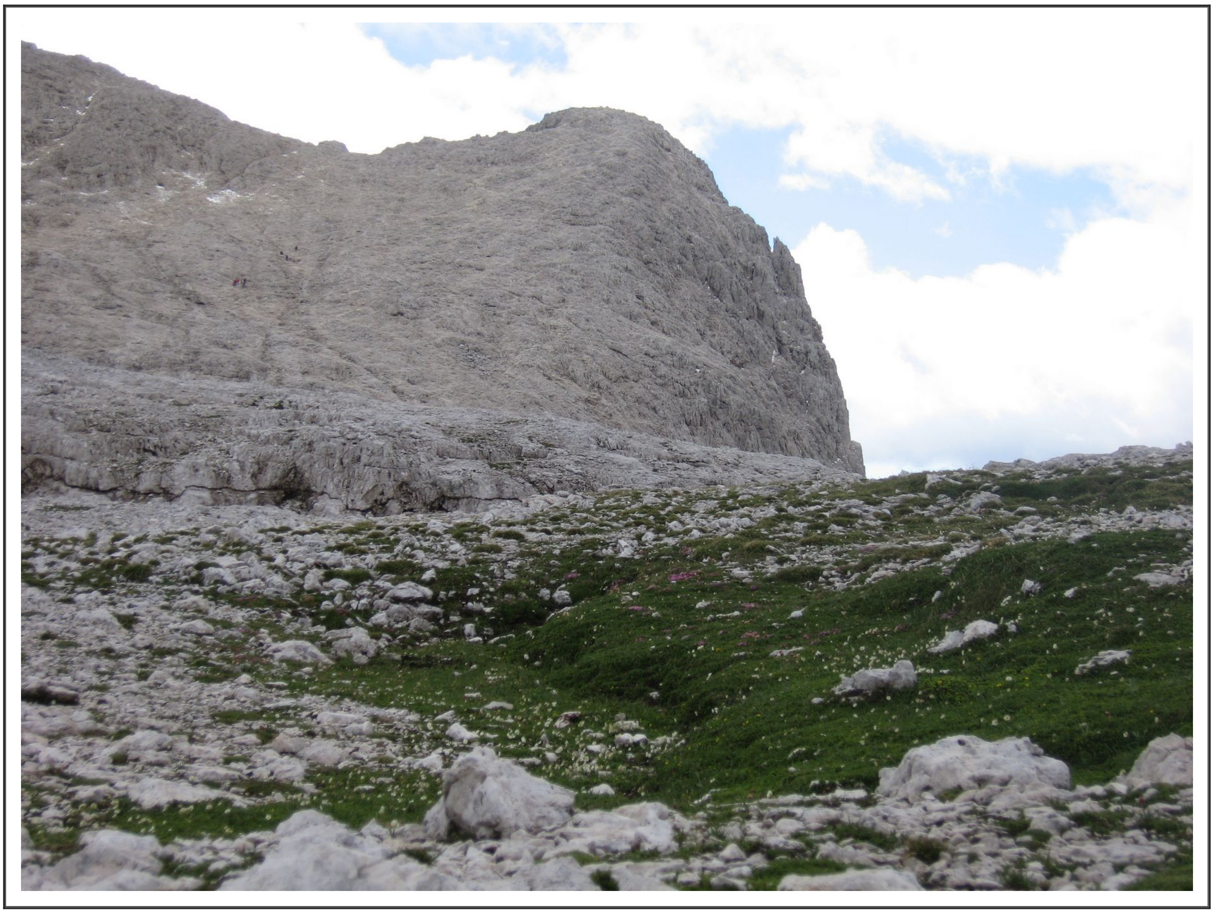

**RS**	Small patches of vegetation on limestone bedrock. NAT2000: 8240, 8120				
altitude m a.s.l.	aspect	slope°	vegetation %	traps	year
2650	E	20°	10%	6	2013

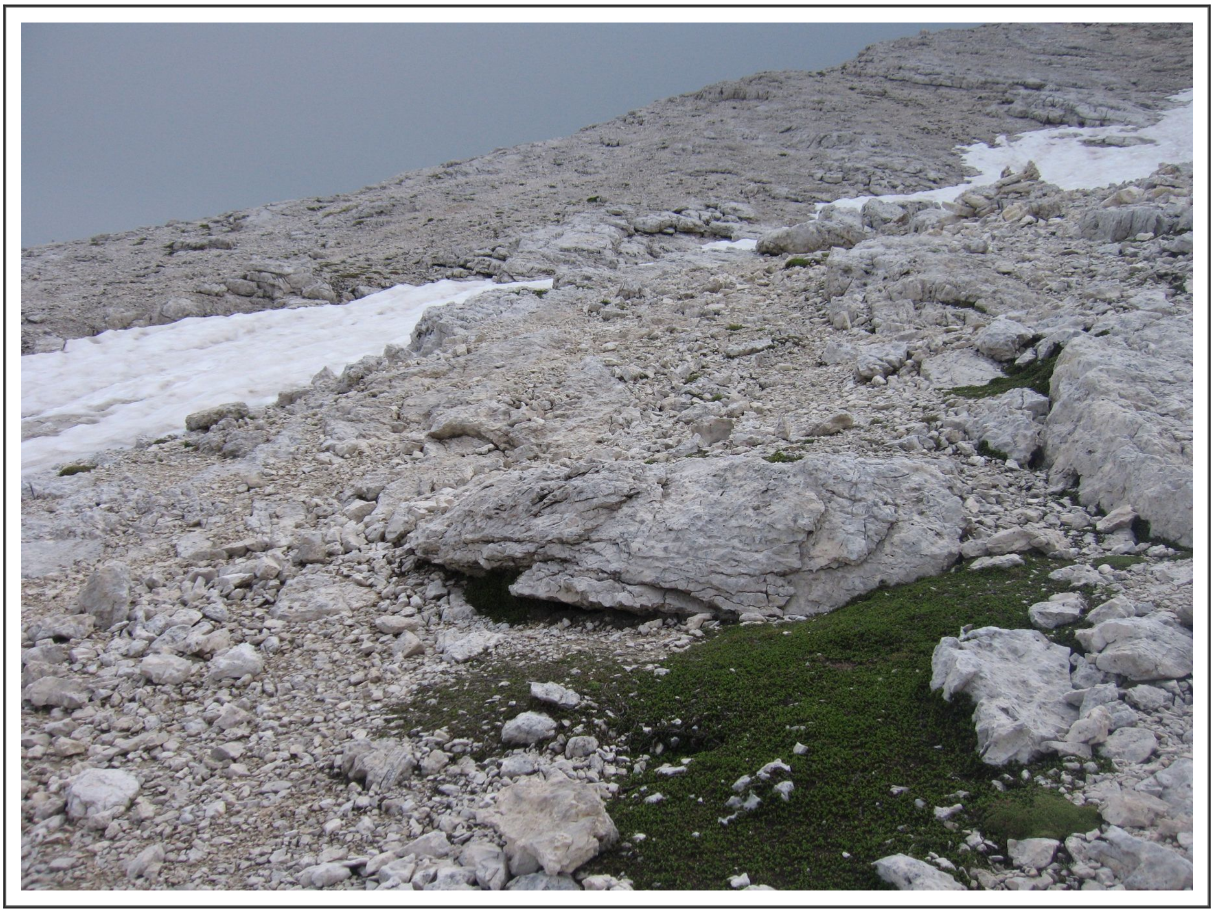

**CF1**	Small patches of vegetation on limestone scree. NAT2000: 8240, 8120				
altitude m a.s.l.	aspect	slope°	vegetation %	traps	year
2575	S	40	40%	6	2013-14

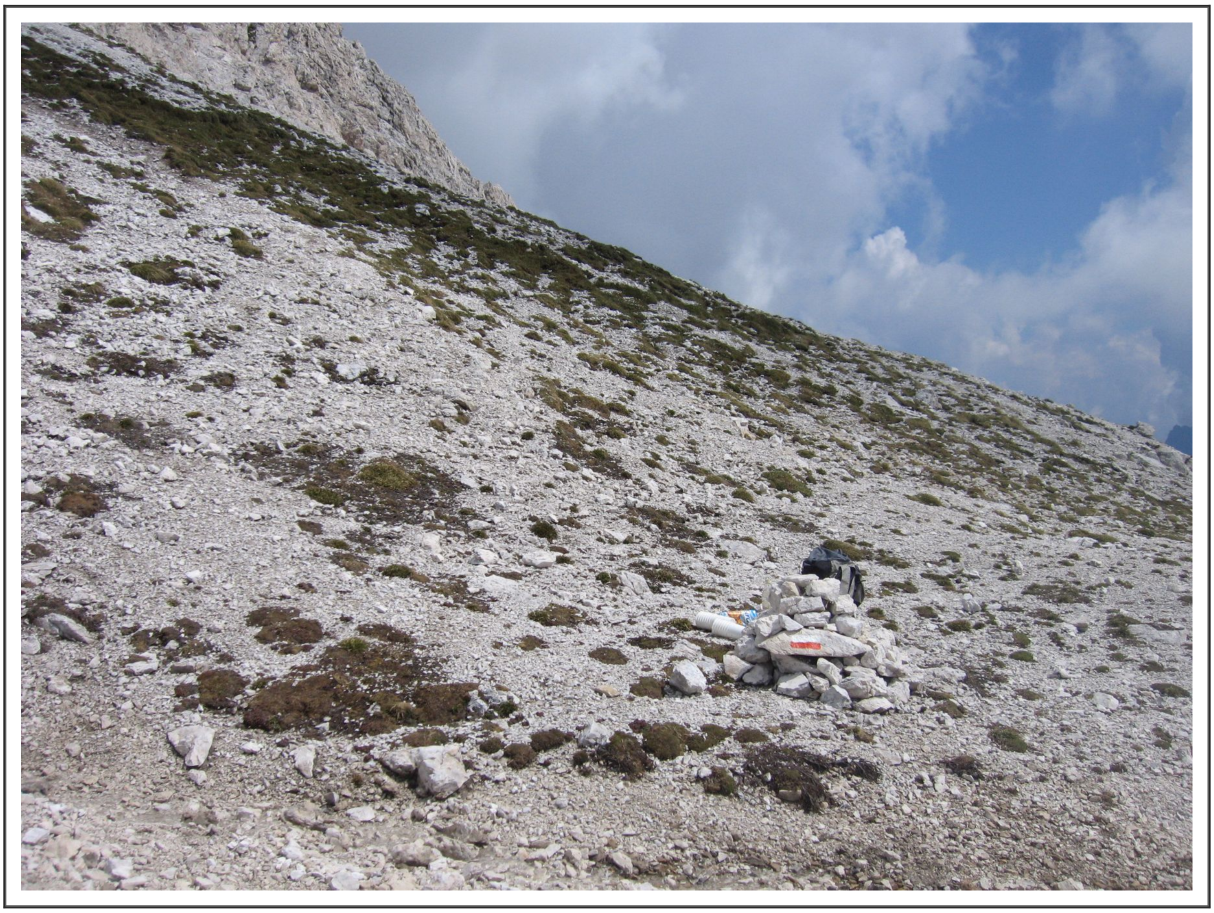

Supplement: Supplementary material 1 — Sampled sites for the dataset on carabid beetles of the Regional Park (Paneveggio e Pale di San Martino, Italy) [file bdj-12-e127417-s001.pdf]
